# Improving Acetate Tolerance of *Escherichia coli* by Rewiring Its Global Regulator cAMP Receptor Protein (CRP)

**DOI:** 10.1371/journal.pone.0077422

**Published:** 2013-10-04

**Authors:** Huiqing Chong, Jianwei Yeow, Ivy Wang, Hao Song, Rongrong Jiang

**Affiliations:** 1 School of Chemical and Biomedical Engineering, Nanyang Technological University, Singapore, Singapore; 2 Life Technologies R&D, Singapore, Singapore; Center for Genomic Regulation, Spain

## Abstract

The presence of acetate exceeding 5 g/L is a major concern during *E. coli* fermentation due to its inhibitory effect on cell growth, thereby limiting high-density cell culture and recombinant protein production. Hence, engineered *E. coli* strains with enhanced acetate tolerance would be valuable for these bioprocesses. In this work, the acetate tolerance of *E. coli* was much improved by rewiring its global regulator cAMP receptor protein (CRP), which is reported to regulate 444 genes. Error-prone PCR method was employed to modify *crp* and the mutagenesis libraries (~3×10^6^) were subjected to M9 minimal medium supplemented with 5–10 g/L sodium acetate for selection. Mutant A2 (D138Y) was isolated and its growth rate in 15 g/L sodium acetate was found to be 0.083 h^-1^, much higher than that of the control (0.016 h^-1^). Real-time PCR analysis via OpenArray^®^ system revealed that over 400 CRP-regulated genes were differentially expressed in A2 with or without acetate stress, including those involved in the TCA cycle, phosphotransferase system, etc. Eight genes were chosen for overexpression and the overexpression of *uxaB* was found to lead to *E. coli* acetate sensitivity.

## Introduction

Acetate, either lignocellulosic-derived or as a fermentative byproduct, can pose a major problem in microbial bioprocesses, especially in the presence of excess glucose [1-6]. Being one of the most widely studied growth inhibitors for *E. coli* fermentations, acetate is known to inhibit cell growth when its concentration exceeds 5 g/L, thereby limiting high cell density and leading to reduced titers or recombinant protein production [7-9]. Therefore, it becomes valuable to engineer strains with improved acetate tolerance for sustainable microbial fermentation [10,11]. The protonated form of acetate can penetrate cell membranes, resulting in a reduction of intracellular pH [9] and an accumulation of anions in cell cytoplasm [12], both of which could contribute to growth inhibition.

In order to resolve the undesirable effects of acetate, fermentation conditions have been optimized to reduce acetate formation, such as controlling glucose feed rate, managing dissolved oxygen level, and using fructose instead of glucose as sole carbon source [13]. Acetate toxicity can also be alleviated *via* the addition of methionine [14], glycine [13], arginine, threonine, and lysine [10].

Reduction in acetate production can also be achieved through the adoption of metabolic engineering tools. Genes related to acetate producing pathways were knocked out [15,16], while heterologous genes have been introduced into *E. coli* to convert acetate to other less harmful byproducts [17]. In addition, directed evolution of homoserine o-succinyltransferase, an enzyme involved in methionine biosynthesis, was also proved to enhance the acetate tolerance of *E. coli* [14].

Besides metabolic engineering approaches, classical strain engineering methods of using UV and evolutionary engineering strategies have also been used to improve microbial tolerance towards acetate stress. UV mutagenesis was performed on *Clostridium thermoaceticum* and *Clostridium thermoautophicum* [18], whereas acetate-tolerant *E. coli* and *S. cerevisiae* mutants were generated through evolutionary engineering [11,19].

Since classical strain engineering approaches are often time- and labour- intensive, and metabolic engineering tools are only limited to a few microorganisms with well-studied metabolic pathways [20-23], new strain engineering approaches such as genome shuffling [24] and transcriptional engineering have been developed to improve strain performance under various stresses. The reported transcription factors include zinc-finger containing artificial transcription factors [25,26], Spt15 [27], sigma factors [28], H-NS [29], Hha [30], and IrrE [31].

Our lab has successfully improved the tolerance of *E. coli* towards various stresses in the past through engineering its global regulator cAMP receptor protein [32-36]. In this work, we have also chosen cAMP-receptor protein (CRP) as our target regulator to improve the acetate tolerance of *E. coli*. To our knowledge, this is the first project on engineering a native global regulator to improve strain acetate tolerance. CRP is able to regulate 444 genes in *E. coli*, especially those involved in carbon metabolism [37,38]. The production and assimilation of acetate is largely controlled by the reactions in the tricarboxylic acid (TCA) cycle, where the genes are mainly regulated by CRP [39,40], which makes CRP an attractive target. Here, we have introduced random mutations to CRP by error-prone PCR. The mutant with the greatest enhancement in acetate tolerance was further characterized in terms of its cross-tolerance to other fermentative byproducts, extracellular acetate concentration and cell morphology studies. The expression changes of 444 CRP-regulated genes were also assessed by quantitative real-time reverse transcription PCR (RT-PCR) using OpenArray^®^ system.

## Materials and Methods

### Materials

The host strain *E. coli* DH5α ∆*crp* was constructed by knocking out *crp* from *E. coli* DH5α (Invitrogen, San Diego, US) according to previously established protocol [41]. Overnight culture was prepared in Luria-Bertani (LB) medium containing 1% tryptone (Oxoid, Hampshire, UK), 0.5% yeast extract (Merck, Damstadt, Germany), and 1% sodium chloride (Merck, Damstadt, Germany). M9 minimal medium was used for cells cultured under acetate stress, which is composed of the following chemicals (per liter): 6.78 g Na_2_HPO_4_, 3 g KH_2_PO_4_, 0.5 g NaCl, 1 g NH_4_Cl, 0.49 g MgSO_4_.7H_2_O, 0.011 g CaCl_2_, 2 g glucose and 1 ml of trace metal stock solution. The trace metal stock solution contained 0.8 g CoCl_2_, 0.4 g ZnSO_4_.7H_2_O, 2 g MnCl_2_.4H_2_O, 0.2 g Na_2_MoO_4_.2H_2_O, 0.2 g CuCl_2_, 2.5 g FeSO_4_.7H_2_O and 1 g thiamine per litre. Sodium acetate was added to M9 minimal medium accordingly when required. All chemicals were purchased from either Merck or Sigma-Aldrich. Restriction enzymes were from Fermentas (Burlington, Canada), while T4 DNA ligase was purchased from New England Biolabs (Ipswich, MA, USA). Gel purification and plasmid extraction were performed using the QIAquick gel extraction kit and the QIAprep spin miniprep kit (QIAGEN, Germany), respectively.

### Library construction

Error-prone PCR was carried out using the GeneMorph® II Random Mutagenesis Kit (Agilent Technologies, Santa Clara, CA, USA) with the following primers: 5’- GAGAGGATCCATAACAGAGGATAACCGCGCATG-3’, and 5’- AGATGGTACCAAACAAAATGGCGCGCTACCAGGTAACGCGCCA-3’ (the underlined sequences correspond to *Bam*HI and *Kpn*I restrictions sites respectively), with 30 ng template to yield 1-3 amino acid substitutions per *crp* gene. The PCR conditions were set as follows: 2 min at 95°C, 30 cycles of 95°C for 1 min, 62°C for 1 min, followed by 72°C for 1 min, and 10 min at 72°C. The resulting *crp* products were digested and cloned into a digested low copy number (copy number 1-5) self-constructed plasmid pKCP (Figure S1 & Table S1) and transformed into *E. coli* DH5α ∆*crp* cells by electroporation using an Eppendorf® multiporator (Hamburg, Germany).

### Mutant selection

The transformants were first cultured in 50 ml M9 minimal medium supplemented with 5 g/L sodium acetate for 20-30 h after inoculation, followed by another two subcultures containing 10 g/L sodium acetate to select for the mutants with improved acetate tolerance. Each subculture was cultivated for ~48 h. The cells were plated onto LB agar plates after each round of selection, and individual clones were randomly picked for sequencing. In order to exclude false positives and spontaneous mutations generated during the selection, the mutated *crp* fragments of the selected plasmids were digested, re-ligated with freshly digested plasmid pKCP and re-transformed into fresh *E. coli* DH5α ∆*crp* background.

### Mutant growth under acetate stress

The growth profile of the CRP mutant isolated from the error-prone PCR libraries was determined at 0, 10 and 15 g/L sodium acetate concentrations. One percent (v/v) overnight cell culture was inoculated into 10-ml M9 medium supplemented with sodium acetate, and cultured at 37°C with orbital shaking at 200 rpm. Their absorbance at 600 nm was recorded at certain time intervals.

### Extracellular acetate concentration

One percent (v/v) overnight cell culture was inoculated either into M9 medium (0 g/L sodium acetate) or M9 medium with 10 g/L sodium acetate. To measure the extracellular acetate concentration, 200-µl cell samples were centrifuged at 13,000 rpm for 5 min, and their supernatant was collected. The acetate concentration was determined enzymatically using a R-biopharm acetic acid test kit (Damstadt, Germany) according to the manufacturer’s instructions.

### Cross-tolerance to other fermentative products

One percent (v/v) overnight cell culture was inoculated into 10-ml M9 medium supplemented with either 5 g/L sodium formate or 18 g/L sodium propionate. Cells were incubated at 37°C with orbital shaking at 200 rpm, and their OD600 values were measured.

### RNA isolation

One percent (v/v) overnight cell culture was inoculated into M9 medium supplemented with or without 10 g/L sodium acetate. Cells were cultured in the absence of sodium acetate for 8 h, or in 10 g/L sodium acetate for 18 h. Total RNA was isolated using the PureLink® RNA mini kit (Life Technologies, Carlsbad, CA, USA), and treated with the PureLink® DNase (Life Technologies, Carlsbad, CA, USA) according to the manufacturer’s instructions. The quality and integrity of the isolated RNA was determined through spectrophotometer and gel electrophoresis. 800 ng total RNA was converted into cDNA by reverse transcription in a 20-µl reaction mixture using the High Capacity cDNA Reverse Transcription Kit (Life Technologies, Carlsbad, CA, USA) with random primer mix following the manufacturer’s protocol.

### RT-PCR using the OpenArray® System

RT-PCR for 444 CRP-regulated genes was conducted with the OpenArray® Real-time PCR instrument (Life Technologies, Carlsbard, CA, USA) in duplicates, using the Lightcycler® (Roche, Germany) FastStart® (Roche, Germany) DNA Master SYBR® (Life Technologies, Carlsbard, CA, USA) Green I kit (Roche, Germany) in the reformatted OpenArray® real-time PCR plates (Life Technologies, Carlsbard, CA, USA). The OpenArray® system is a high throughput real-time PCR platform, which allows 224 genes to be assessed on a single plate. 33-nl reaction mixture was loaded into each through-hole on the plates with the OpenArray® AccuFill^TM^ system (Life Technologies, Carlsbard, CA, USA). The default real-time PCR conditions in the provided protocol were followed. The bacterial 16S rRNA (*rrsG*) was used as the internal standard and the sequence of the primers are given in Table S2. The values of cycle threshold (C_t_) were provided by the OpenArray® Real-time qPCR Analysis Software Version 1.0.4. The 2^-ΔΔCt^ method was utilized to compute the relative expression level. The *p*-value for each gene was calculated by student’s *t*-test using the IBM SPSS Statistics Software Version 19.

### RT-PCR for genes involved in acetate production

The expression level for genes involved in acetate production (*ackA*, *pta* and *poxB*) was measured in the StepOnePlus^TM^ real-time PCR system (Life Technologies, Carlsbard, CA, USA) using the Power SYBR® Green PCR Master Mix (Life Technologies, Carlsbad, CA, USA). The real-time PCR conditions were carried out as follows: 10 min at 95°C, 40 cycles of 95°C for 15 s, followed by 60°C for 1 min. The bacterial 16S rRNA (*rrsG*) was used as an internal standard and the sequences of the primers are provided in Table S2. The values of cycle threshold (C_t_) were provided by the software, and 2^-ΔΔCt^ method of relative quantification was utilized to compute the relative expression level. The *p*-value for each gene was calculated by student’s *t*-test.

### Effects of gene overexpression on acetate tolerance

The ASKA clones (GFP-) was used for gene overexpression [42] in *E. coli* DH5α. One percent (v/v) overnight culture was inoculated into 10-ml M9 medium containing either 0 g/L or 5 g/L sodium acetate. Chloramphenicol (25 µg/ml) was added to maintain the pCA24N-based plasmids, and 0.1 mM IPTG was used to induce the overexpression of genes of interest according to previously established protocol [29]. Cells were incubated at 37°C with orbital shaking at 200 rpm, and their OD600 values were recorded.

## Results and Discussion

### Mutant isolation

The mutated *crp* products obtained *via* error-prone PCR were cloned into plasmid pKCP. *E. coli* DH5α ∆*crp* strain harboring pKCP (containing native *crp* operon), which was used as the control in this study. Six error-prone PCR libraries with a total library size of ~3×10^6^ were subjected to 5-10 g/L sodium acetate for variant selection. The mutant selection process was greatly shortened to 5-7 days as compared to classical strain engineering methods of using adaptive evolution whereby 1,750 generations (~18 days) were required to isolate an acetate-tolerant mutant [19]. Thirty-two colonies were randomly picked and sequenced to determine their amino acid modifications in CRP. Among these 32 clones, eight of them contained mutation Y63F (A1), eight had D138Y (A2), ten contained V176I (A3), and the remaining six clones had modifications at Y99C, A151V, V176A, and L195M (A4). A2 was identified with the best improvement in acetate tolerance out of these four variants and was investigated further in this study.

The amino acid substitution at position D138 of A2 (D138Y) is of particular interest as some other CRP mutants with either improved osmotolerance or 1-butanol tolerance also had this D138 modification [32,35], but unlike A2, those mutants also carried modifications at other locations. D138 is known to play essential roles during the hinge reorientation and helical adjustment upon allosteric activation by cAMP [43] through the formation of hydrogen bonds with G141 [44]. Although D138 is not located in the DNA binding domain (residue 140-209), any modifications to this position may affect the DNA binding affinity of CRP [45].

### Mutant growth under acetate stress

**Figure 1:**
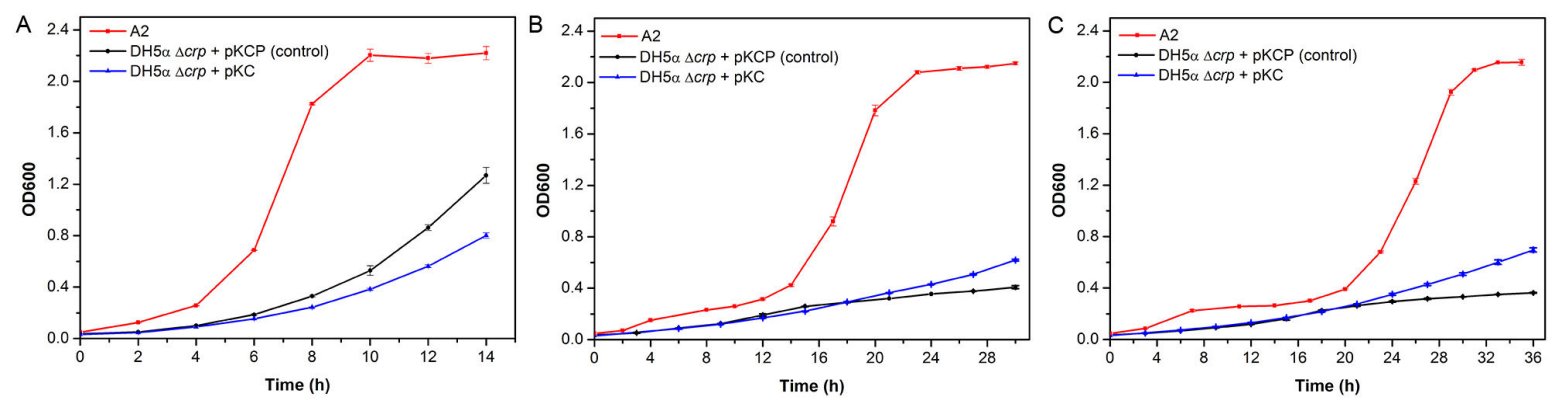
Growth profile. Mutant strain A2, the control (DH5α Δ*crp* harbouring plasmid pKCP containing native *crp* operon) and DH5α Δ*crp* strain harbouring blank plasmid pKC in M9 medium at 37 °C (**A**) 0 g/L sodium acetate (**B**) 10 g/L sodium acetate (**C**) 15 g/L sodium acetate. Average values and standard deviations were calculated from triplicate experiments.

The growth profiles of A2 and the control were determined in the absence or presence of acetate stress. When cells were grown in M9 medium without sodium acetate supplement (Figure 1A), it is found that A2 (0.19 ± 0.002 h^-1^) already outgrew the control (0.13 ± 0.002 h^-1^). The presence of 10 g/L (Figure 1B) and 15 g/L sodium acetate (Figure 1C) led to even stronger growth inhibition of the control. Although the growth rate of A2 was significantly higher than the control under acetate stress, the presence of acetate resulted in A2 exhibiting long period of lag phase up to 20 h. With 10 g/L sodium acetate present, the growth rate of A2 was 0.10 ± 0.0004 h^-1^, higher than that of the control at 0.026 ± 0.001 h^-1^. When sodium acetate concentration was further increased to 15 g/L, the growth rate of A2 became 0.083 ± 0.0003 h^-1^, whereas that of the control dropped to 0.016 ± 0.001 h^-1^.

### Extracellular acetate concentration

**Figure 2:**
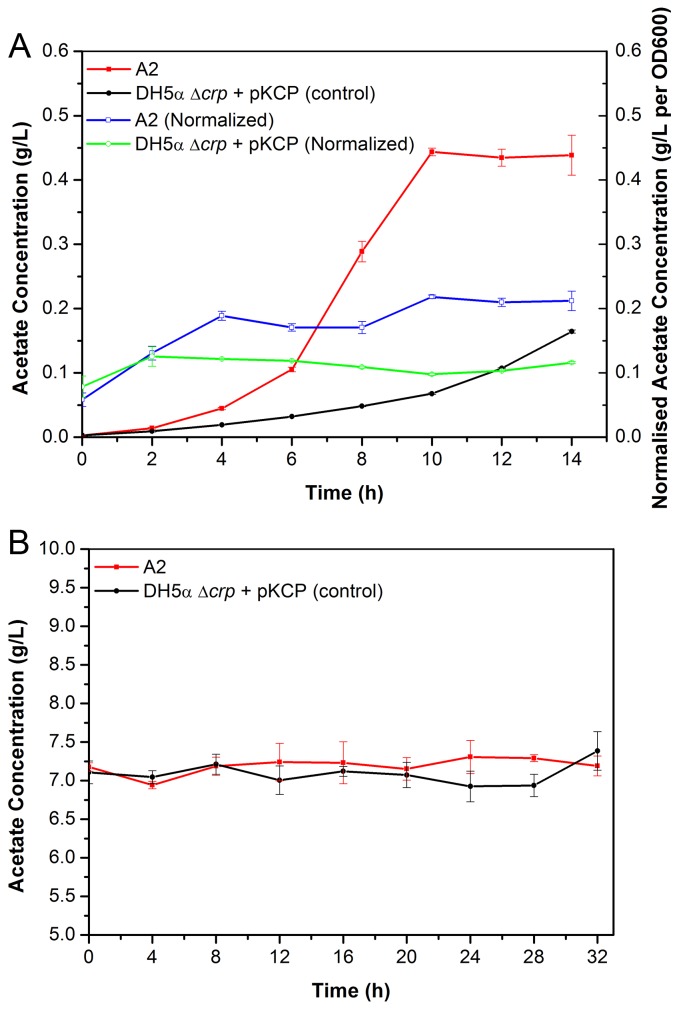
Extracellular acetate concentration of A2 and the control. (**A**) M9 medium (0 g/L sodium acetate), normalized by cell OD600 values and (**B**) M9 minimal medium supplemented with 10 g/L sodium acetate. Average values and standard deviations were calculated from triplicate experiments.

The amount of acetate produced by A2 and the control were monitored during cultivation. Without sodium acetate supplement, it was observed that the acetate produced by A2 was higher than the control (Figure 2A). Since the cell growth of A2 was also higher than the control during cultivation, the measured acetate concentration was normalized by OD600 values. The normalized acetate concentration of A2 (average 0.17 g/L per OD600) almost doubled that of the control (average 0.10 g/L per OD600) over the sampling period of 14 h. On the contrary, when A2 and the control were exposed to 10 g/L sodium acetate (~7 g/L acetate), the extracellular acetate concentration was nearly the same (Figure 2B).

It was reported before that strains with less acetate production would grow faster [46], thus many strategies have been focusing on reducing the production of acetate to improve cell growth under acetate stress [47]. Interestingly, we found that although A2 was able to grow faster than the control in minimal medium, A2 produced more acetate than the control. There are two acetate producing pathways in *E. coli*: the first is from acetyl-CoA to acetate catalyzed by acetate kinase (*ackA*) and phosphotransacetylase (*pta*) with acetyl-phosphate as intermediate, and the second is to convert pyruvate to acetate by pyruvate kinase (*poxB*) [48]. As demonstrated by our RT-PCR results on *ackA*, *pta* and *poxB* (Figure S2), A2 had higher expression of all three genes than the control. Therefore, the boosted acetate production in A2 was likely to be attributed to the higher expression of these three genes.

### Cross-tolerance to other fermentative byproducts

**Figure 3:**
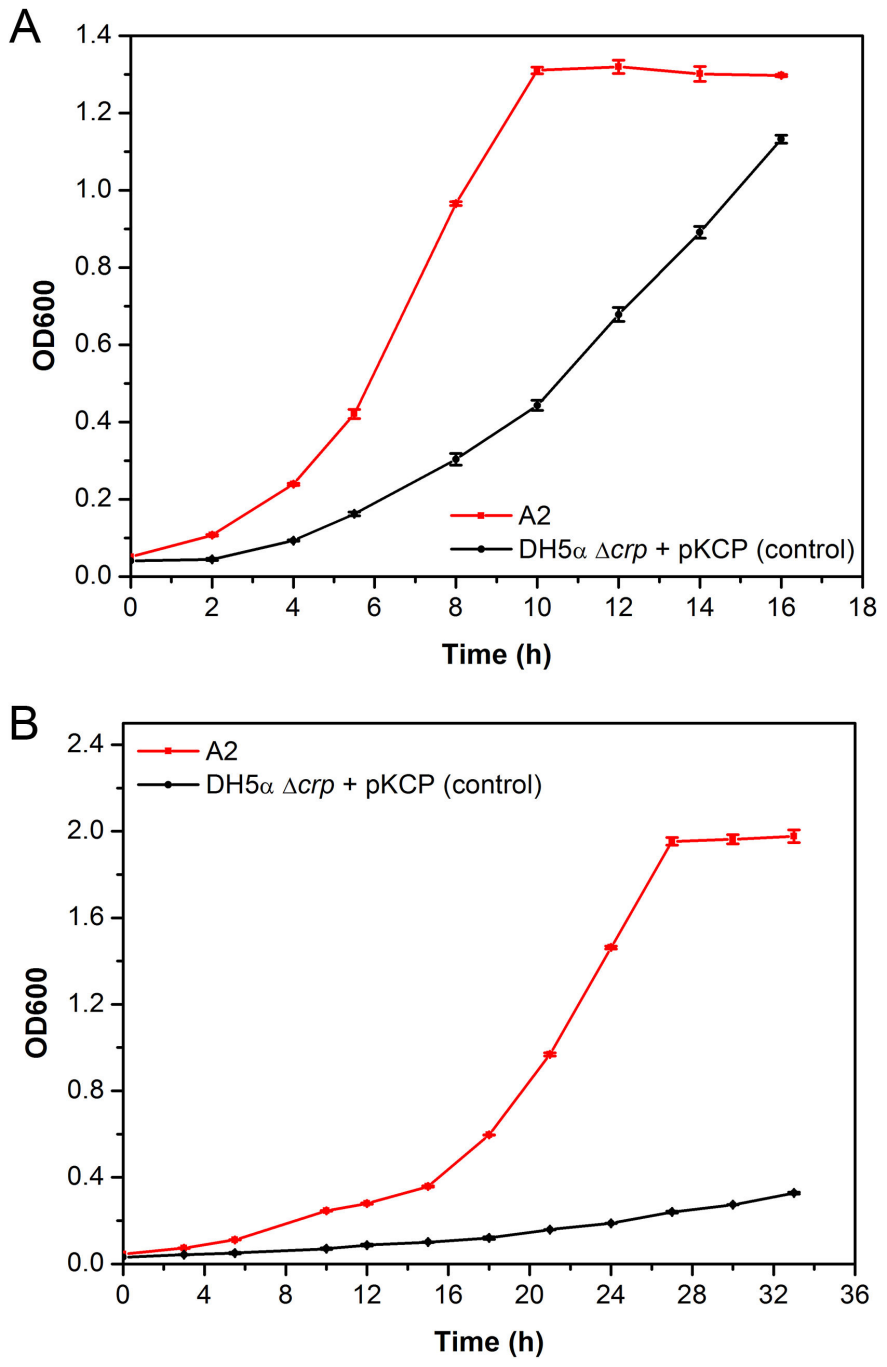
Cell growth of variant A2 and the control in the presence of (A) 5 g/L sodium formate (B) 18 g/L sodium propionate in M9 medium, 37 °C. Average values and standard deviations were calculated from triplicate experiments.

In addition to acetate, other metabolic byproducts, such as formate and propionate, are present during *E. coli* fermentation [8,49]. Hence, the tolerance of A2 towards these two byproducts was also investigated. The growth pattern of A2 and the control cultivated in M9 medium supplemented with 5 g/L sodium formate or 18 g/L sodium propionate was presented in Figure 3A and 3B respectively. The growth rate of A2 in formate was 0.16 ± 0.002 h^-1^, higher than the control (0.14 ± 0.008 h^-1^), and its performance was much enhanced in the presence of propionate—the growth rate of A2 was 0.07 ± 0.0004 h^-1^, doubling that of the control at 0.03 ± 0.0003 h^-1^.

### Quantitative real-time reverse transcription PCR with an OpenArray® system

We have investigated on the relative expression level of 444 genes regulated by CRP, about 10% of genes in *E. coli* [37], between mutant A2 and the control in the presence or absence of sodium acetate stress. This was performed to probe the difference of the transcript level of CRP-regulated genes between A2 and the control. Without stress, 407 genes were differentially expressed in A2, including 290 up-regulated genes and 14 down-regulated genes by more than 2-fold (*p* < 0.05) (Table S3). The two highest upregulated genes were *yfiD* and *pflB*, by 17.7- and 15.5-fold respectively, whereas *galE* displayed the leading downregulation of 143-fold. On the other hand, the presence of sodium acetate stress resulted in the differential expression of 432 CRP-regulated genes in A2, with 6 upregulated and 406 genes downregulated by more than 2-fold at a *p*-value threshold < 0.05 (Table S4). To be more specific, *galE* had the biggest increase in A2 by nearly 14-fold, whereas *araG*, encoding the ATP binding unit of arabinose transporter [37], exhibited the greatest decrease of 27-fold.

It is apparent that the presence of sodium acetate resulted in the downregulation of various functional groups in A2, such as the TCA cycle (*sdhCDAB*, *sucABCD*, and *mdh*), phosphotransferase system (PTS) (*ptsG*, *ptsHI-crr*, *nagE*, and *malX*), ATP binding cassette (ABC) transporters (*malEFG*, *mglBAC*, *flhCD*, and *lsrB*), two-component system (*ompF*, *dctA*), amino acid metabolism (*tnaA*, *gadA*), and amino sugar and nucleotide sugar metabolism (*nanE*) (Table 1). Many of these genes have already been demonstrated to be associated with cell adaptation to acetate stress [39,49,50]. Interestingly, except for the aforementioned genes involved in the TCA cycle, the rest of the genes were all upregulated when sodium acetate was absent. The downregulation of TCA cycle in the absence of sodium acetate may lead to high acetate accumulation in A2 [47].

**Table 1:** Selected CRP-regulated genes with differential expression in A2 as compared to the control from various metabolic pathways (*p* < 0.05).

**Metabolic Pathway**	**Gene or operon**	Fold-change* (A2/control)	
		-sodium acetate	+sodium acetate
Pentose and glucuronate interconversions	*uxaB*	3.2	-22
TCA cycle	*sdhCDAB*	-1.7 to -3.8	-12.5 to -13.6
	*sucABCD*	-2.7 to -5.1	-4.3 to -7.3
	*mdh*	-2.2	-3
Galactose metabolism	*galE*	-143	14.3
Glyoxylate pathway	*aceB* and *aceA*	-2 to -2.7	-6.4 to -7
	*aceK*	2	-8.7
	*acnB*	-3.2	-6.5
Phosphotransferase system (PTS)	*ptsG*	1.1	-5.5
	*ptsHI-crr*	1.3 to 1.7	-1.3 to -2.9
	*nagE*	2.4	-10.3
	*malX*	1.9	-15
Pyruvate metabolism	*acs*	-	-7.4
	*pflB*	15.5	4.4
	*yfiD*	17.7	2.1
ABC Transporters	*malEFG*	2.2 to 3.9	-9.3 to -12.2
	*mglBAC*	2.0 to 2.2	-11.8 to -14.4
	*flhCD*	2.0 to 2.2	-11.3 to -12.6
	*lsrB*	2.4	-23.8
Two-component system	*ompF*	-4.0	-10.5
	*dctA*	2.6	-10.1
Tryptophan metabolism	*tnaA*	3.3	-8.1
Alanine, aspartate and glutamate metabolism	*gadA*	2.6	3.1
Amino sugar and nucleotide sugar metabolism	*nanE*	-	-6.1

* Fold-change and statistics were calculated from duplicate samples.

Acetate can also be utilised as carbon source, whereby the uptake of acetate is performed by acetyl-CoA synthetase (acs), and metabolized through the glyoxylate cycle (*aceBAK* operon, *mdh*, *gltA* and *acnB*). Many studies on the response of *E. coli* towards acetate stress have reported that *acs* and *aceBAK* are induced by acetate [46]. However, as presented in Table 1, we found that the expression of *acs* was downregulated by over 7-fold, and the genes from glyoxylate pathway had repressed expression in A2 with acetate stress present.

Acetate can lead to internal acidification whereby the dissociation of weak acids in cytoplasm causes decline in intracellular pH [12,51]. Genes such as *yfiD*, *pflB*, *gadA* and *gadB* are able to increase their expression to protect *E. coli* against internal acidification (Table 1) [50,52], which concurs with our findings in this study. In particular, *yfiD*, which encodes stress-induced alternate pyruvate formate lyase subunit, was upregulated by almost 17-fold even without the addition of sodium acetate. The expression of *pflB* (pyruvate formate lyase, 15.5-fold), *gadA* (2.6-fold) and *gadB* (glutamate decarboxylase, 2.3-fold) also enhanced in A2, suggesting that A2 was protected against internal acidification by the pre-programmed upregulation of these genes. The upregulation of *yfiD, pflB* and *gadA* (approximately 2- to 3-fold) in the presence of sodium acetate might ensure the continual protection of A2 against internal acidification caused by acetate anion. Yet, we found that the overexpression of individual genes such as *pflB*, *yfiD*, or *gadA* did not confer acetate tolerance to *E. coli* (data not shown), indicating that coordinated changes from multiple genes might be required [53].

Previous DNA microarray analysis of *E. coli* after long-term adaption to acetate stress illustrated that certain genes involved in the uptake and catabolism of carbon and energy sources, except for glucose, decreased in their mRNA abundances, such as maltose (*malXY*, and *malEFG*), galactose (*galETKM*, *galS*, and *mglBAC*), amino sugars (*nanATEK-yhcH*), sugar alcohols (*srlA1A2BD-gutM*, and *srlR-gutQ*), amino acids (*tnaA*), and dicarboxylic acids (*dctA*) to adapt to acetate stress [49]. Our results on A2 also display repression of these genes under acetate stress. Another similar finding is that the expression of transport genes such as *malE* and *ompF* are both repressed in the presence of acetate [8,50].

### Effects of gene overexpression on acetate tolerance

**Figure 4:**
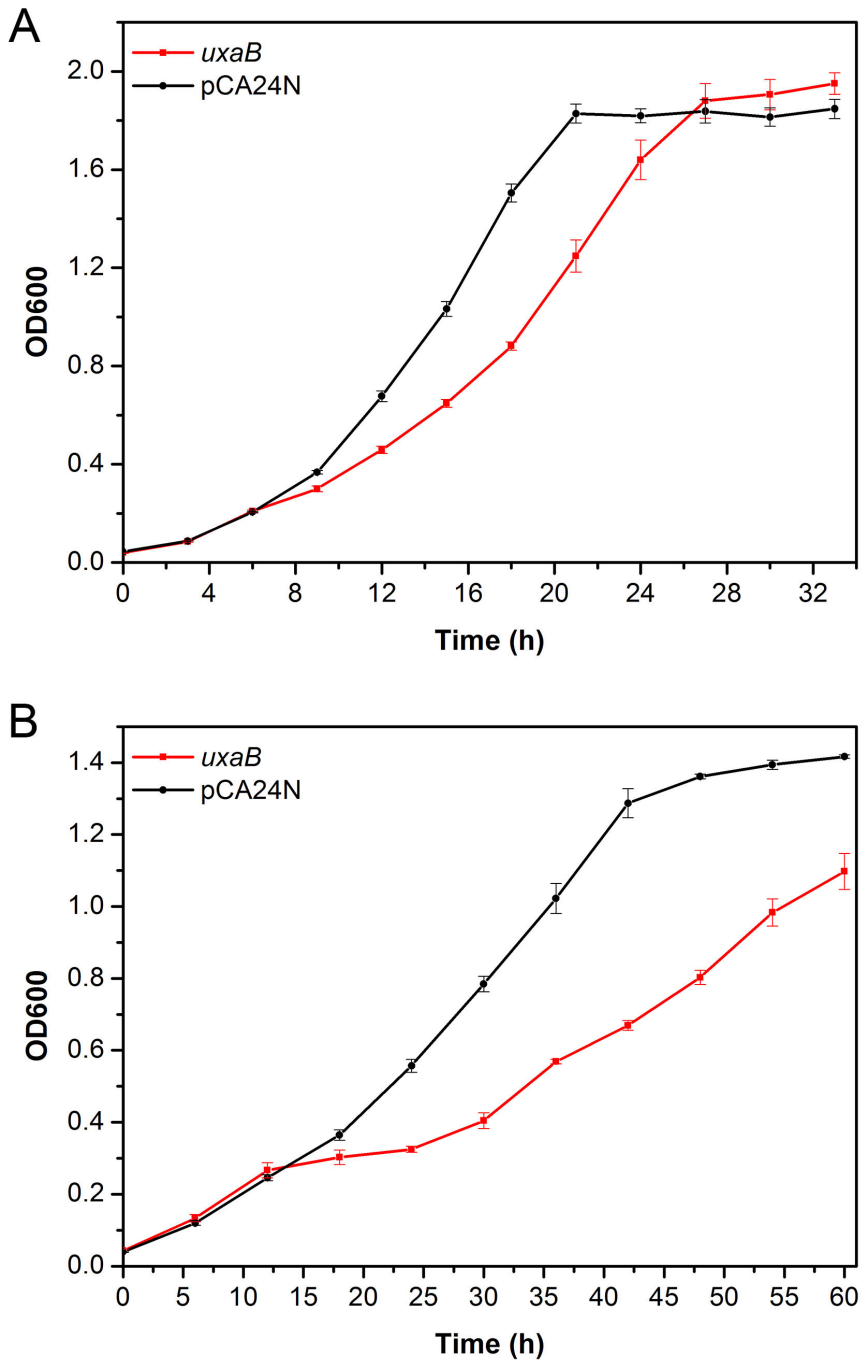
Cell growth of *E. coli* DH5α overexpressing *uxaB* in M9 medium, with *E. coli* DH5α harbouring pCA24N as control. (**A**) 0 g/L sodium acetate (**B**) 5 g/L sodium acetate. Average values and standard deviations were calculated from triplicate experiments.

Eight genes with the largest fold-change in their expression level in A2 under acetate stress (Table S4), including the upregulated genes *pflB*, *yfiD*, *galE*, *gadA* (2- to 14.3-fold), and the downregulated genes *uxaB*, *feaR* and *araG* (-22- to -27.3-fold), were chosen for overexpression. *E. coli* DH5α harboring pCA24N plasmid was adopted as control. It was observed that the overexpression of *pflB*, *yfiD*, *galE* and *gadA* did not improve the growth of *E. coli* DH5α when facing stress, and the overexpression of *feaR* and *araG* did not result in acetate sensitivity (data not shown). In contrast, a noticeable growth difference appeared between the control and *uxaB* overexpression in the presence of acetate stress. Although both had similar growth rate of 0.09-0.1 h^-1^ when cultured without sodium acetate (Figure 4A), the presence of 5g/L sodium acetate reduced the growth of the control to ~ 0.05 h^-1^ and exerted a greater deleterious effect on the cells overexpressing *uxaB* (Figure 4B)—its cell growth rate was reduced by ~60% to 0.021 h^-1^. *uxaB* (altronate oxidoreductase) is responsible for the reversible NADH-dependent reduction of D-tagaturonate to D-altronate in galacturonate catabolism pathway [54]. To our knowledge, there have been no reports to date relating *uxaB* with acetate metabolism or tolerance, which may require further investigation in the future.

## Conclusions

In this study, we have successfully isolated an *E. coli* mutant strain with much enhanced tolerance towards acetate stress by rewiring its global regulator CRP. Error-prone PCR was adopted to introduce mutations to CRP and the mutant selection process was greatly shortened to several days as compared to classical strain engineering methods of using adaptive evolution. To the extent of our knowledge, this is the first study whereby a native regulator is engineered by random mutagenesis method for improved cell performance under acetate stress.

## Supporting Information

Figure S1 **Vector map of plasmid pKCP.** The plasmid contains native promoter and terminator of the *crp* operon.
(TIF)

Figure S2 **Fold-change in the expression level of selected genes.** A2 (grey) and the control (white) when cultivated in M9 minimal medium supplemented with (**A**) 0 g/L sodium acetate and (**B**) 10 g/L sodium acetate. * *p* < 0.05, # *p* < 0.01 and ** *p* < 0.001, compared to the control using *t*-test (mean ± standard deviation, n = 3).
(TIF)

Table S1 **DNA sequence of plasmid pKCP.** The plasmid contains native promoter and terminator of the *crp* operon.
(DOC)

Table S2 **Primers used in OpenArray® real time PCR.**
(DOC)

Table S3 **CRP-regulated genes with >2-fold change in their expression level in A2 as compared to the control in the absence of sodium acetate stress, using a *p*-value threshold less than 0.05.**
(DOC)

Table S4 **CRP-regulated genes with >2-fold change in their expression level in A2 as compared to the control in the presence of sodium acetate stress, using a *p*-value threshold less than 0.05.**
(DOC)
