# Supplementary figures and images for: Improving Acetate Tolerance of Escherichia coli by Rewiring Its Global Regulator cAMP Receptor Protein (CRP)
Source: PLoS One. 2013 Oct 4;8(10):e77422. doi: 10.1371/journal.pone.0077422 (PMC3790751; doi:10.1371/journal.pone.0077422)

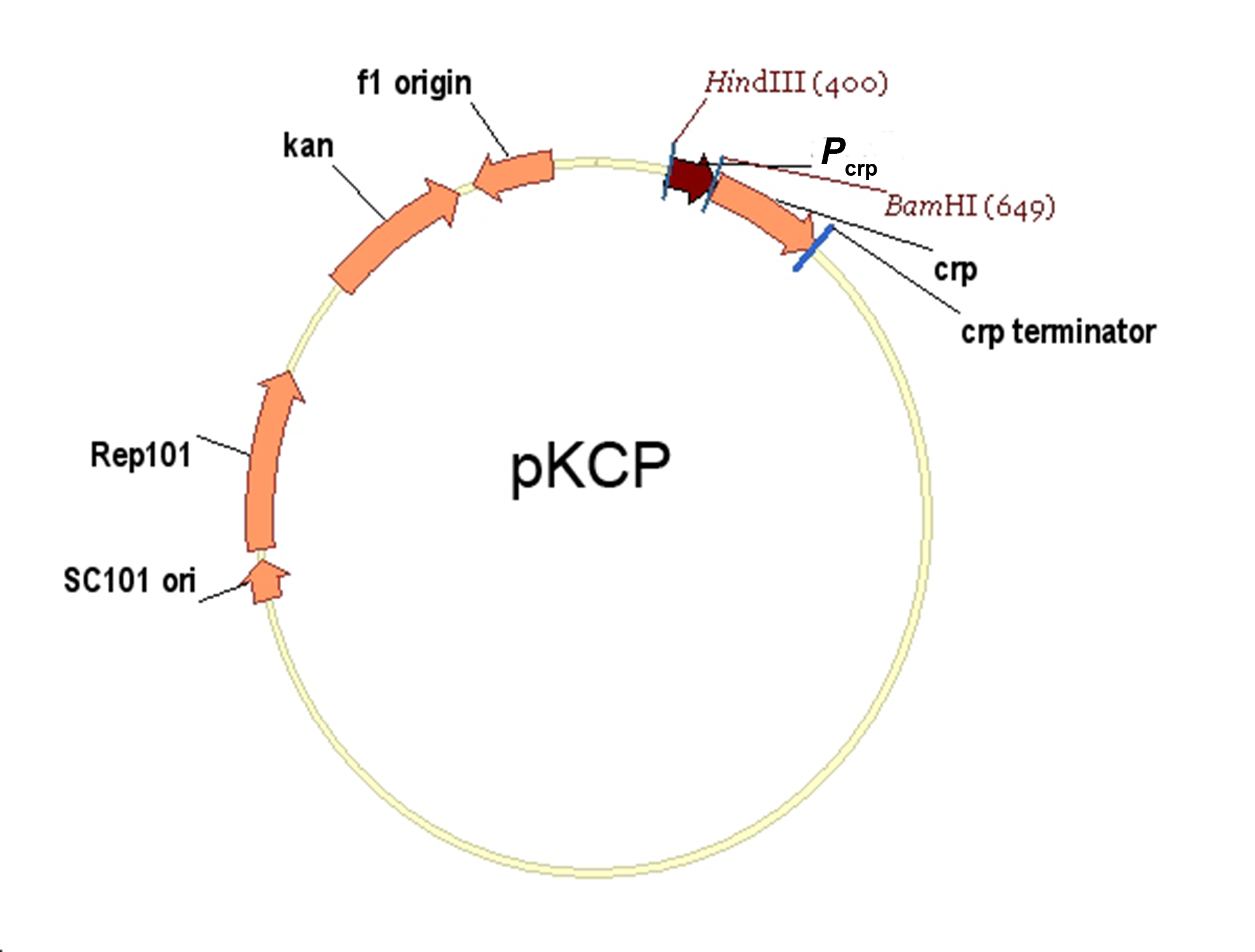

Supplement: Figure S1 — Vector map of plasmid pKCP. The plasmid contains native promoter and terminator of the crp operon. (TIF) [file pone.0077422.s001.tif]

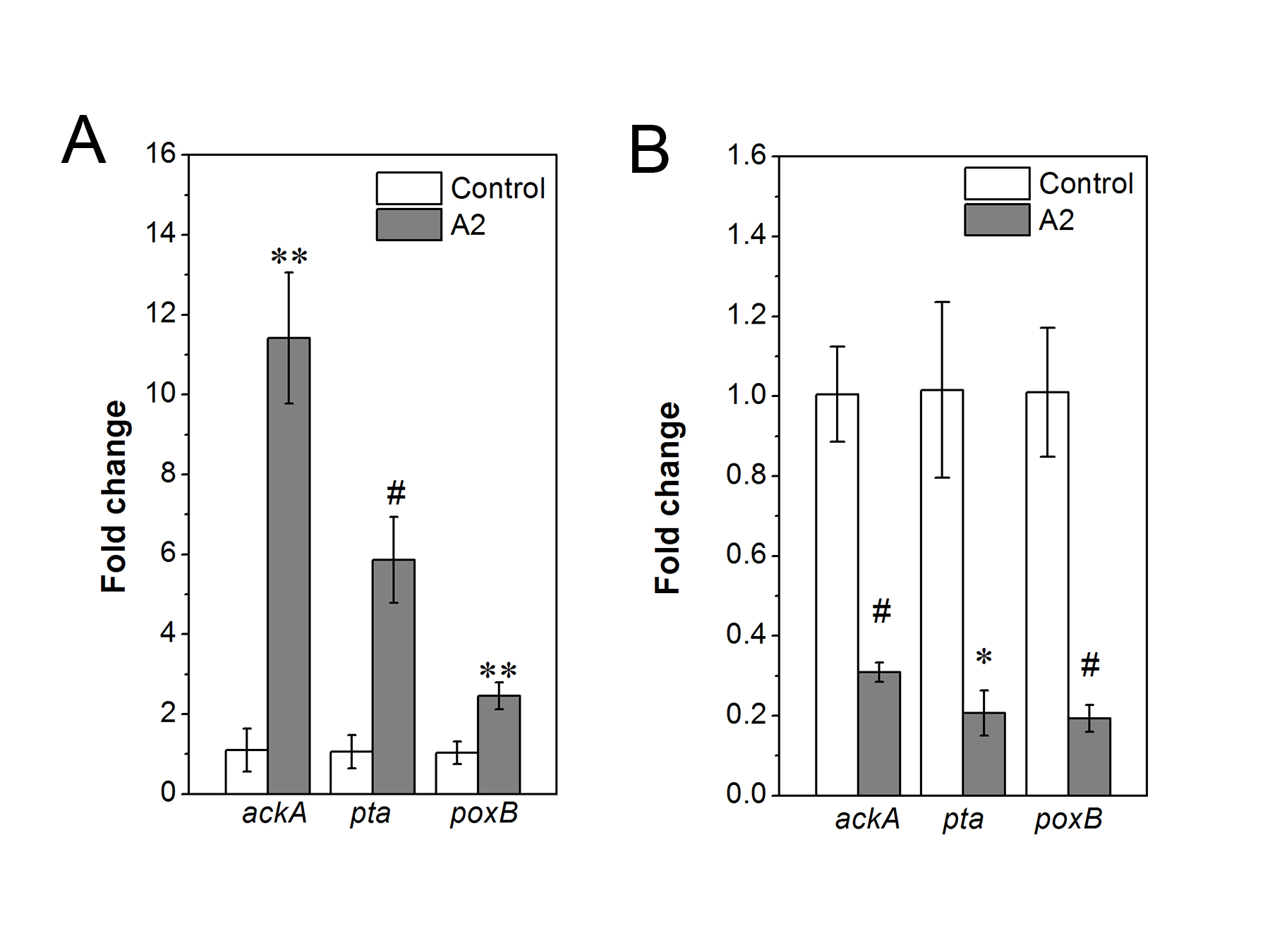

Supplement: Figure S2 — Fold-change in the expression level of selected genes. A2 (grey) and the control (white) when cultivated in M9 minimal medium supplemented with (A) 0 g/L sodium acetate and (B) 10 g/L sodium acetate. * p < 0.05, # p < 0.01 and ** p < 0.001, compared to the control using t-test (mean ± standard deviation, n = 3). (TIF) [file pone.0077422.s002.tif]
