# Supplementary material for: Improving Acetate Tolerance of Escherichia coli by Rewiring Its Global Regulator cAMP Receptor Protein (CRP)
Source: PLoS One. 2013 Oct 4;8(10):e77422. doi: 10.1371/journal.pone.0077422 (PMC3790751; doi:10.1371/journal.pone.0077422)
Supplement: Table S1 — DNA sequence of plasmid pKCP. The plasmid contains native promoter and terminator of the crp operon. (DOC) [file pone.0077422.s003.doc]

1 TCGCGCGTTT CGGTGATGAC GGTGAAAACC TCTGACACAT GCAGCTCCCG GAGACGGTCA

61 CAGCTTGTCT GTAAGCGGAT GCCGGGAGCA GACAAGCCCG TCAGGGCGCG TCAGCGGGTG

121 TTGGCGGGTG TCGGGGCTGG CTTAACTATG CGGCATCAGA GCAGATTGTA CTGAGAGTGC

181 ACCATATGCG GTGTGAAATA CCGCACAGAT GCGTAAGGAG AAAATACCGC ATCAGGCGCC

241 ATTCGCCATT CAGGCTGCGC AACTGTTGGG AAGGGCGATC GGTGCGGGCC TCTTCGCTAT

301 TACGCCAGCT GGCGAAAGGG GGATGTGCTG CAAGGCGATT AAGTTGGGTA ACGCCAGGGT

361 TTTCCCAGTC ACGACGTTGT AAAACGACGG CCAGTGCCAA GCTTAGAGTA CGCGTACTAA

421 CCAAATCGCG CAACGGAAGG CGACCTGGGT CATGCTGAAG CGAGACACCA GGAGACACAA

481 AGCGAAAGCT ATGCTAAAAC AGTCAGGATG CTACAGTAAT ACATTGATGT ACTGCATGTA

541 TGCAAAGGAC GTCACATTAC CGTGCAGTAC AGTTGATAGC CCCTTCCCAG GTAGCGGGAA

601 GCATATTTCG GCAATCCAGA GACAGCGGCG TTATCTGGCT CTGGAGAGGA TCCATAACAG

661 AGGATAACCG CGCATGGTGC TTGGCAAACC GCAAACAGAC CCGACTCTCG AATGGTTCTT

721 GTCTCATTGC CACATTCATA AGTACCCATC CAAGAGCACG CTTATTCACC AGGGTGAAAA

781 AGCGGAAACG CTGTACTACA TCGTTAAAGG CTCTGTGGCA GTGCTGATCA AAGACGAAGA

841 GGGTAAAGAA ATGATCCTCT CCTATCTGAA TCAGGGTGAT TTTATTGGCG AACTGGGCCT

901 GTTTGAAGAG GGCCAGGAAC GTAGCGCATG GGTACGTGCG AAAACCGCCT GTGAAGTGGC

961 TGAAATTTCG TACAAAAAAT TTCGCCAATT GATTCAGGTA AACCCGGACA TTCTGATGCG

1021 TTTGTCTGCA CAGATGGCGC GTCGTCTGCA AGTCACTTCA GAGAAAGTGG GCAACCTGGC

1081 GTTCCTCGAC GTGACGGGCC GCATTGCACA GACTCTGCTG AATCTGGCAA AACAACCAGA

1141 CGCTATGACT CACCCGGACG GTATGCAAAT CAAAATTACC CGTCAGGAAA TTGGTCAGAT

1201 TGTCGGCTGT TCTCGTGAAA CCGTGGGACG CATTCTGAAG ATGCTGGAAG ATCAGAACCT

1261 GATCTCCGCA CACGGTAAAA CCATCGTCGT TTACGGCACT CGTTAATCCC GTCGGAGTGG

1321 CGCGTTACCT GGTAGCGCCC CATTTTGTTT GGTACCGAAG AACCGTGGTA TGTGGTGGAC

1381 AAAGACGTCG AAAACAACAT TCTGGTTGTC GCTCAGGGCC ATGAACACCC GCGGCTGATG

1441 TCTGTCGGGT TGATTGCCCA GCAGTTGCAC TGGGTCGATC GCGAACCATT CACCGGCACT

1501 ATGCGTTGCA CGGTAAAAAC CCGCTATCGC CAGACCGATA TCCCTTGCAC CGTCAAGGCG

1561 CTGGACGATG ATCGCATTGA AGTGATTTTC GATGAACCGG TTGCCGCCGT GACGCCGGGC

1621 CAGTCTGCCG TCTTCTATAA CGGTGAAGTG TGCCTCGGTG GCGGTATTAT TGAGCAGCGT

1681 CTGCCGCTGC CGGTCTGATT ATTATCTTTA CTTAAAACAA GGAAGCAGTG AACGTGGCAA

1741 AGAATTACTA TGACATCACC CTCGCCCTGG CCGGTATTTG TCAGTCGGCA CGCCTGGTGC

1801 AACAACTCGC TCACCAGGGG CATTGTGATG CCGATGCGCT ACACGTCTCA CTCAACAGTA

1861 TTATTGATAT GAACCCCAGC TCGACGCTGG CGGTTTTTGG CGGTAGCGAA GCCAACCTGC

1921 GCGTCGGGCT GGAAACCCTG CTCGGCGTGC TCAATGCCAG CAGTCGCCAG GGCTTAAACG

1981 CCGAATTAAC CCGCTACACA CTCAGCTTGA TGGTGCTTGA GCGCAAACTC TCCTCAGCGA

2041 AAGGCGCGCT CGACACTCTG GGCAACCGGA TCAACGGCCT GCAACGCCAG CTCGAACACT

2101 TCGATTTACA GTCCGAAACG CTGATGAGCG CGATGGCTGC TATCTATGTT GATGTGATTA

2161 GCCCGCTTGG CCCGCGCATT CAGGTCACCG GTTCCCCTGC TGTACTGCAA AGCCCACAAG

2221 TGCAGGCGAA AGTTCGCGCA ACCCTGCTGG CAGGCATTCG CGCCGCCGTG CTCTGGCACC

2281 AGGTCGGCGG CGGACGTCTG CAACTGATGT TTTCTCGTAA TCGCCTGACC ACTCAGGCAA

2341 AACAAATTCT TGCTCATTTA ACCCCGGAGT TGTGATCTAT GGAATTATCC TCACTGACCG

2401 CCGTTTCCCC TGTCGATGGA CGCTACGGCG ATAAAGTCAG CGCGCTGCGC GGGATTTTCA

2461 GCGAATATGG TTTGCTGAAA TTCCGTGTAC AAGTTGAAGT ACGTTGGCTG CAAAAACTGG

2521 CCGCGCACGC AGCGATCAAG GAAGTTCCTG CTTTTGCTGC CGACGCAATC GGTTACCTTG

2581 ATGCAATCGT CGCCAGTTTC AGCGAAGAAG ATGCGGCGCG CATCAAAACT ATCGAGCGTA

2641 CCACTAACCA CGACGTTAAA GCGGTTGAGT ATTTCCTGAA AGAAAAAGTG GCGGAGATCC

2701 CGGAACTGCA CGCGGTTTCT AAATTCATCC ACTTTGCCTG TACTTCGGAA GATATCAATA

2761 ACCTCTCCCA CGCATTAATG CTGAAAACCG CGCGTGATGA AGTGATCCTG CCATACTGGC

2821 GTCAACTGAT TGATGGCATT AAAGATCTCG CCGTTCAGTA TCGCGATATC CCGCTGCTGT

2881 CTCGTACCCA CGGTCAGCCA GCCACGCCGT CAACCATCGG TAAAGAGATG GCAAACGTCG

2941 CCTACCGTAT GGAGCGCCAG TACCGCCAGC TTAACCAGGT GGAGATCCTC GGCAAAATCA

3001 ACGGCGCGGT CGGTAACTAT AACGCCCACA TCGCCGCTTA CCCGGAAGTT GACTGGCATC

3061 AGTTCAGCGA AGAGTTCGTC ACCTCGCTGG GTATTCAGTG GAACCCGTAC ACCACCCAGA

3121 TCGAACCGCA CGACTACATT GCCGAACTGT TTGATTGCGT TGCGCGCTTC AACACTATTC

3181 TGATCGACTT TGACCGTGAC GTCTGGGGTT ATATCGCCCT TAACCACTTC AAACAGAAAA

3241 CCATTGCTGG TGAGATTGGT TCTTCCACCA TGCCGCATAA AGTTAACCCG ATCGACTTCG

3301 AAAACTCCGA AGGGAATCTG GGCCTTTCCA ACGCGGTATT GCAGCATCTG GCAAGCAAAC

3361 TGCCGGTTTC CCGCTGGCAG CGTGACCTGA CCGACTCTAC CGTGCTGCGT AACCTCGGCG

3421 TGGGTATCGG TTATGCCTTG ATTGCATATC AATCCACCCT GAAAGGCGTG AGCAAACTGG

3481 AAGTGAACCG TGACCATCTG CTGGATGAAC TGGATCACAA CTGGGAAGTG CTGGCTGAAC

3541 CAATCCAGAC AGTTATGCGT CGCTATGGCA TCGAAAAACC GTACGAGAAG CTGAAAGAGC

3601 TGACTCGCGG TAAGCGCGTT GACGCCGAAG GCATGAAGCA GTTTATCGAT GGTCTGGCGT

3661 TGCCAGAAGA AGAGAAAGCC CGCCTGAAAG CGATGACGCC GGCTAACTAT ATTGGTCGAG

3721 CTATCACGAT GGTTGATGAG CTGAAATAAA CCTCGTATCA GTGCCGGATG GCGATGCTGT

3781 CCGGCCTGCT TATTAAGATT ATCCGCTTTT TATTTTTTCA CTTTACCTCC CCTCCCCGCT

3841 GGTTTATTTA ATGTTTACCC CCATAACCAC ATAATCGCGT TACACTATTT TAATAATTAA

3901 GACAGGGAGA AATAAAAATG CGCGTACTGG TTGTTGAAGA CAATGCGTTG TTACGTCACC

3961 ACCTTAAAGT TCAGATTCAG GATGCTGGTC ATCAGGTCGA TGACGCAGAA GATGCCAAAG

4021 AAGCCGATTA TTATCTCAAT GAACATATAC CGGATATTGC GATTGTCGAT CTCGGATTGC

4081 CAGACGAGGA CGGTCTGTCA CTGATTCGCC GCTGGCGTAG CAACGATGTT TCACTGCCGA

4141 TTCTGGTATT AACCGCCCGT GAAAGCTGGC AGGACAAAGT CGAAGTATTA AGTGCCGGTG

4201 CTGATGATTA TGTGACTAAA CCGTTTCATA TTGAAGAGGT GATGGCGCGA ATGCAGGCAT

4261 TAATGCGGCG TAATAGCGGT CTGGCTTCAC AGGTCATTTC GCTCCCCCCG TTTCAGGTTG

4321 ATCTCTCTCG CCGTGAATTA TCTATTAATG ACGAAGTGAT CAAACTGACC GCGTTCGAAT

4381 ACACCATTAT GGAAACGTTG ATACGCAATA ATGGCAAAGT GGTCAGCAAA GATTCGTTAA

4441 TGCTCCAACT CTATCCGGAT GCGGAGCTGC GGGAAAGCCA TACCATTGAT GTACTGATGG

4501 GACGTCTGCG CAAAAAAATT CAGGCACAAT ATCCCCAAGA AGTGATTACC ACCGTTCGCG

4561 GCCAGGGCTA TCTGTTCGAA TTGCGCTGAT GAAAAAATTA CTGCGTCTTT TTTTCCCGCT

4621 CTCGCTGCGG GTACGTTTTC TGTTGGCAAC GGCAGCGGTA GTACTGGTGC TTTCGCTTGC

4681 CTACGGAATG GTCGCGCTGA TCGGTTATAG CGTCAGTTTC GATAAAACTA CGTTTCGGCT

4741 GTTACGTGGC GAGAGCAATC TGTTCTATAC CCTTGCGAAG TGGGAAAACA ATAAGTTGCA

4801 TGTCGAGTTA CCCGAAAATA TCGACAAGCA AAGCCCCACC ATGACGCTAA TTTATGATGA

4861 GAACGGGCAG CTTTTATGGG CGCAACGTGA CGTGCCCTGG CTGATGAAGA TGATCCAGCC

4921 TGACTGGCTG AAATCGAATG GTTTTCATGA AATTGAAGCG GATGTTAACG ATACCAGCCT

4981 CTTGCTGAGT GGAGATCATT CGATACAGCA ACAGTTGCAG GAAGTGCGGG AAGATGATGA

5041 CGACGCGGAG ATGACCCACT CGGTGGCAGT AAACGTCTAC CCGGCAACAT CGCGGATGCC

5101 AAAATTAACC ATTGTGGTGG TGGATACCAT TCCGGTGGAG CTAAAAAGTT CCTATATGGT

5161 CTGGAGCTGG TTTATCTATG TGCTCTCAGC CAATCTGCTG TTAGTGATCC CGCTGCTGTG

5221 GGTCGCCGCC TGGTGGAGTT TACGCCCCAT CGAAGCCCTG GCAAAAGAAG TCCGCGAACT

5281 GGAAGAACAT AACCGCGAAT TGCTCAATCC AGCCACAACG CGAGAACTGA CCAGTCTGGT

5341 ACGAAACCTG AACCGATTGT TAAAAAGTGA ACGCGAACGT TACGACAAAT ACCGTACGAC

5401 GCTCACCGAC CTGACCCATA GTCTGAAAAC GCCACTGGCG GTGCTGCAAA GTACGCTGCG

5461 TTCTCTGCGT AGTGAAAAGA TGAGCGTCAG TGATGCTGAG CCGATAATGC TGGAGCAAAT

5521 CAGCCGCATT TCACAGCAAA TTGGCTACTA CCTGCATCGT GCCAGTATGC GCGGCGGGAC

5581 ATTGCTCAGC CGCGAGCTGC ATCCGGTCGC CCCACTGCTG GACAATCTCA CCTCAGCGCT

5641 GAACAAAGTG TATCAACGCA AAGGGGTCAA TATCTCTCTC GATATTTCGC CAGAGATCAG

5701 CTTTGTCGGT GAGCAGAACG ATTTTGTCGA GGTGATGGGC AACGTGCTGG ATAATGCCTG

5761 TAAATATTGC CTCGAGTTTG TCGAAATTTC TGCAAGGCAA ACCGACGAGC ATCTCTATAT

5821 TGTGGTCGAG GATGATGGCC CCGGTATTCC ATTAAGCAAG CGAGAGGTCA TTTTCGACCG

5881 TGGTCAACGG GTTGATACTT TACGCCCTGG GCAAGGTGTA GGGCTGGCGG TAGCCCGCGA

5941 AATCACCGAG CAATATGAGG GTAAAATCGT CGCCGGAGAG AGCATGCTGG GCGGTGCGCG

6001 GATGGAGGTG ATTTTTGGTC GCCAGCATTC TGCGCCGAAA GATGAATAAA TATGCCCATA

6061 CTTCACGCAT TACGTTAAGC ATCCGTTATA ATCGGTTGCA GATACCAGCC TGTGGATGCT

6121 TAACATGGAA TACCAACTCA CTCTTAACTG GCCCGATTTT CTTGAACGTC ACTGGCAGAA

6181 ACGCCCGGTG GTGTTAAAAC GCGGCTTTAA TAATTTTATT GACCCGATCT CTCCAGACGA

6241 GTTGGCGGGT CTGGCGATGG AAAGCGAAGT TGACAGTCGA CTGGTCAGTC ACCAGGATGG

6301 CAAATGGCAG GTCAGCCACG GCCCGTTCGA AAGCTACGAT CATCTCGGTG AAACCAACTG

6361 GTCATTACTG GTACAGGCAG TGAACCACTG GCATGAGCCG ACCGCCGCGC TGATGCGACC

6421 GTTCCGTGAA CTACCGGACT GGCGTATTGA TGATCTGATG ATTTCTTTTT CTGTACCCGG

6481 CGGCGGCGTC GGCCCGCATC TCGATCAGTA CGACGTGTTT ATCATTCAGG GTACCCAGGC

6541 ATCAAATAAA ACGAAAGGCT CAGTCGGAAG ACTGGGCCTT TCGTTTTATC TGTTGTTTGT

6601 CGGTGAACGC TCTCCTGAGT AGGACAAATC CGCCGGGAGC GGATTTGAAC GTTGCGAAGC

6661 AACGGCCCGG AGGGTGGCGG GCAGGACGCC CGCCATAAAC TGCCAGGCAT CAAATTAAGC

6721 AGAAGGCCAT CCTGACGGAT GGCCTTTTTG CGTTTCTACA AACTCTTCCT GTCGTCATAT

6781 CTACAGAATT CGTAATCATG GTCATAGCTG TTTCCTGTGT GAAATTGTTA TCCGCTCACA

6841 ATTCCACACA ACATACGAGC CGGAAGCATA AAGTGTAAAG CCTGGGGTGC CTAATGAGTG

6901 AGCTAACTCA CATTAATTGC GTTGCGCTCA CTGCCCGCTT TCCAGTCGGG AAACCTGTCG

6961 TGCCAGCTGC ATTAATGAAT CGGCCAACGC GCGGGGAGAG GCGGTTTGCG TATTGGGCGC

7021 TCTTCCGCTT CCTCGCTCAC TGACTCGCTG CGCTCGGTCG TTCGGCTGCG GCGAGCGGTA

7081 TCAGCTCACT CAAAGGCGGT AATACGGTTA TCCACAGAAT CAGGGGATAA CGCAGGAAAG

7141 AACATGGACG TGTCAGCGGC TTTCCAGAAC TCGGACGGTT TATGCGCTGC CCACGCCGGC

7201 ATATTGCCGG ACTCCTTGTG CTCAAGGTCG GAGTCTTTTT CACGGGCATA CTTTCCCTCA

7261 CGCGCAATAT AATCGGCATG AGGAGAGGCA CTGCCTTTTC CGCCGGTTTT TACGCTGAGA

7321 TGATAGGATG CCATCGTGTT TTATCCCGCT GAAGGGCGCA CGTTTCTGAA CGAAGTGAAG

7381 AAAGTCTAAG TGCGCCCTGA TAAATAAAAG AGTTATCAGG GATTGTAGTG GGATTTGACC

7441 TCCTCTGCCA TCATGAGCGT AATCATTCCG TTAGCATTCA GGAGGTAAAC AGCATGAATA

7501 AAAGCGAAAA AACAGGAACA ATGGGCAGCA GAAAGAGTGC AGTATATTCG CGGCTTAAAG

7561 TCGCCGAATG AGCAACAGAA ACTTATGCTG ATACTGACGG ATAAAGCAGA TAAAACAGCA

7621 CAGGATATCA AAACGCTGTC CCTGCTGATG AAGGCTGAAC AGGCAGCAGA GAAAGCGCAG

7681 GAAGCCAGAG CGAAAGTCAT GAACCTGATA CAGGCAGAAA AGCGAGCCGA AGCCAGAGCC

7741 GCCCGTAAAG CCCGTGACCA TGCTCTGTAC CAGTCTGCCG GATTGCTTAT CCTGGCGGGT

7801 CTGGTTGACA GTAAGACGGG TAAGCCTGTT GATGATACCG CTGCCTTACT GGGTGCATTA

7861 GCCAGTCTGA ATGACCTGTC ACGGGATAAT CCGAAGTGGT CAGACTGGAA AATCAGAGGG

7921 CAGGAACTGC TGAACAGCAA AAAGTCAGAT AGCACCACAT AGCAGACCCG CCATAAAACG

7981 CCCTGAGAAG CCCGTGACGG GCTTTTCTTG TATTATGGGT AGTTTCCTTG CATGAATCCA

8041 TAAAAGGCGC CTGTAGTGCC ATTTACCCCC ATTCACTGCC AGAGCCGTGA GCGCAGCGAA

8101 CTGAATGTCA CGAAAAAGAC AGCGACTCAG GTGCCTGATG GTCGGAGACA AAAGGAATAT

8161 TCAGCGATTT GCCCGAGCTT GCGAGGGTGC TACTTAAGCC TTTAGGGTTT TAAGGTCTGT

8221 TTTGTAGAGG AGCAAACAGC GTTTGCGACA TCCTTTTGTA ATACTGCGGA ACTGACTAAA

8281 GTAGTGAGTT ATACACAGGG CTGGGATCTA TTCTTTTTAT CTTTTTTTAT TCTTTCTTTA

8341 TTCTATAAAT TATAACCACT TGAATATAAA CAAAAAAAAC ACACAAAGGT CTAGCGGAAT

8401 TTACAGAGGG TCTAGCAGAA TTTACAAGTT TTCCAGCAAA GGTCTAGCAG AATTTACAGA

8461 TACCCACAAC TCAAAGGAAA AGGACTAGTA ATTATCATTG ACTAGCCCAT CTCAATTGGT

8521 ATAGTGATTA AAATCACCTA GACCAATTGA GATGTATGTC TGAATTAGTT GTTTTCAAAG

8581 CAAATGAACT AGCGATTAGT CGCTATGACT TAACGGAGCA TGAAACCAAG CTAATTTTAT

8641 GCTGTGTGGC ACTACTCAAC CCCACGATTG AAAACCCTAC AAGGAAAGAA CGGACGGTAT

8701 CGTTCACTTA TAACCAATAC GCTCAGATGA TGAACATCAG TAGGGAAAAT GCTTATGGTG

8761 TATTAGCTAA AGCAACCAGA GAGCTGATGA CGAGAACTGT GGAAATCAGG AATCCTTTGG

8821 TTAAAGGCTT TGAGATTTTC CAGTGGACAA ACTATGCCAA GTTCTCAAGC GAAAAATTAG

8881 AATTAGTTTT TAGTGAAGAG ATATTGCCTT ATCTTTTCCA GTTAAAAAAA TTCATAAAAT

8941 ATAATCTGGA ACATGTTAAG TCTTTTGAAA ACAAATACTC TATGAGGATT TATGAGTGGT

9001 TATTAAAAGA ACTAACACAA AAGAAAACTC ACAAGGCAAA TATAGAGATT AGCCTTGATG

9061 AATTTAAGTT CATGTTAATG CTTGAAAATA ACTACCATGA GTTTAAAAGG CTTAACCAAT

9121 GGGTTTTGAA ACCAATAAGT AAAGATTTAA ACACTTACAG CAATATGAAA TTGGTGGTTG

9181 ATAAGCGAGG CCGCCCGACT GATACGTTGA TTTTCCAAGT TGAACTAGAT AGACAAATGG

9241 ATCTCGTAAC CGAACTTGAG AACAACCAGA TAAAAATGAA TGGTGACAAA ATACCAACAA

9301 CCATTACATC AGATTCCTAC CTACATAACG GACTAAGAAA AACACTACAC GATGCTTTAA

9361 CTGCAAAAAT TCAGCTCACC AGTTTTGAGG CAAAATTTTT GAGTGACATG CAAAGTAAGT

9421 ATGATCTCAA TGGTTCGTTC TCATGGCTCA CGCAAAAACA ACGAACCACA CTAGAGAACA

9481 TACTGGCTAA ATACGGAAGG ATCTGAGGTT CTTATGGCTC TTGTATCTAT CAGTGAAGCA

9541 TCAAGACTAA CAAACAAAAG TAGAACAACT GTTCACCGTT ACATATCAAA GGGAAAACTG

9601 TCCATATGCA CAGATGAAAA CGGTGTAAAA AAGATAGATA CATCAGAGCT TTTACGAGTT

9661 TTTGGTGCAT TCAAAGCTGT TCACCATGAA CAGATCGACA ATGTAACAGA TGAACAGCAT

9721 GTAACACCTA ATAGAACAGG TGAAACCAGT AAAACAAAGC AACTAGAACA TGAAATTGAA

9781 CACCTGAGAC AACTTGTTAC AGCTCAACAG TCACACATAG ACAGCCTGAA ACAGGCGATG

9841 CTGCTTATCG AATCAAAGCT GCCGACAACA CGGGAGCCAG TGACGCCTCC CGTGGGGAAA

9901 AAATCATCTC GAGGAAGATC CTTTGATCTT TTCTACGGGG TCTGACGCTC AGTGGAACGA

9961 AAACTCACGT TAAGGGATTT TGGTCATGAA CAATAAAACT GTCTGCTTAC ATAAACAGTA

10021 ATACAAGGGG TGTTATGAGC CATATTCAAC GGGAAACGTC TTGCTCTAGG CCGCGATTAA

10081 ATTCCAACAT GGATGCTGAT TTATATGGGT ATAAATGGGC TCGCGATAAT GTCGGGCAAT

10141 CAGGTGCGAC AATCTATCGA TTGTATGGGA AGCCCGATGC GCCAGAGTTG TTTCTGAAAC

10201 ATGGCAAAGG TAGCGTTGCC AATGATGTTA CAGATGAGAT GGTCAGACTA AACTGGCTGA

10261 CGGAATTTAT GCCTCTTCCG ACCATCAAGC ATTTTATCCG TACTCCTGAT GATGCATGGT

10321 TACTCACCAC TGCGATCCCC GGGAAAACAG CATTCCAGGT ATTAGAAGAA TATCCTGATT

10381 CAGGTGAAAA TATTGTTGAT GCGCTGGCAG TGTTCCTGCG CCGGTTGCAT TCGATTCCTG

10441 TTTGTAATTG TCCTTTTAAC AGCGATCGCG TATTTCGTCT CGCTCAGGCG CAATCACGAA

10501 TGAATAACGG TTTGGTTGAT GCGAGTGATT TTGATGACGA GCGTAATGGC TGGCCTGTTG

10561 AACAAGTCTG GAAAGAAATG CATAAACTTT TGCCATTCTC ACCGGATTCA GTCGTCACTC

10621 ATGGTGATTT CTCACTTGAT AACCTTATTT TTGACGAGGG GAAATTAATA GGTTGTATTG

10681 ATGTTGGACG AGTCGGAATC GCAGACCGAT ACCAGGATCT TGCCATCCTA TGGAACTGCC

10741 TCGGTGAGTT TTCTCCTTCA TTACAGAAAC GGCTTTTTCA AAAATATGGT ATTGATAATC

10801 CTGATATGAA TAAATTGCAG TTTCATTTGA TGCTCGATGA GTTTTTCTAA GAATTAATTC

10861 ATGAGCGGAT ACATATTTGA ATGTATTTAG AAAAATAAAC AAATAGGGGT TCCGCGCACA

10921 TTTCCCCGAA AAGTGCCACC TGAAATTGTA AGCGTTAATA TTTTGTTAAA ATTCGCGTTA

10981 AATTTTTGTT AAATCAGCTC ATTTTTTAAC CAATAGGCCG AAATCGGCAA AATCCCTTAT

11041 AAATCAAAAG AATAGACCGA GATAGGGTTG AGTGTTGTTC CAGTTTGGAA CAAGAGTCCA

11101 CTATTAAAGA ACGTGGACTC CAACGTCAAA GGGCGAAAAA CCGTCTATCA GGGCGATGGC

11161 CCACTACGTG AACCATCACC CTAATCAAGT TTTTTGGGGT CGAGGTGCCG TAAAGCACTA

11221 AATCGGAACC CTAAAGGGAG CCCCCGATTT AGAGCTTGAC GGGGAAAGCC GGCGAACGTG

11281 GCGAGAAAGG AAGGGAAGAA AGCGAAAGGA GCGGGCGCTA GGGCGCTGGC AAGTGTAGCG

11341 GTCACGCTGC GCGTAACCAC CACACCCGCC GCGCTTAATG CGCCGCTACA GGGCGCGTCC

11401 CATTCGCCAA TCCGGATATA GTTCCTCCTT TCAGCAAAAA ACCCCTCAAG ACCCGTTTAG

11461 AGGCCCCAAG GGGTTATGCT AGTTATTGCT CAGCGGTGGC AGCAGCCAAC TCAGCTTCCT

11521 TTCGGGCTTT GTTTAGCAGC CTAGGTATTA ATCAAGACGT CTAAGAAACC ATTATTATCA

11581 TGACATTAAC CTATAAAAAT AGGCGTATCA CGAGGCCCTT TCGTC
