# Supplementary material for: Improving Acetate Tolerance of Escherichia coli by Rewiring Its Global Regulator cAMP Receptor Protein (CRP)
Source: PLoS One. 2013 Oct 4;8(10):e77422. doi: 10.1371/journal.pone.0077422 (PMC3790751; doi:10.1371/journal.pone.0077422)
Supplement: Table S2 — Primers used in OpenArray® real time PCR. (DOC) [file pone.0077422.s004.doc]

| Gene | Forward sequence (5'🡪3') | Reverse sequence (5'🡪3') |
| --- | --- | --- |
| *aaeA* | GGTTGCGCTGGTGAAACAGAA | CCCTGCGGCAACACTATCAAC |
| *aaeB* | GCTGACGCTGATTACCCAATCC | ATAACTCTCCGCAGGCGTTTGA |
| *aaeR* | TAAACCGTAGCACACGCAGCAT | TGAAGAACAGCCAATGCGTAGC |
| *aceA* | GGCGAGGGTATGAAGCACTACG | ATTCTTCAGTGGAGCCGGTCAG |
| *aceB* | GCTGGAAGCCAATAACGGTCAC | GCATACCTTCTTCGGTGCGTTC |
| *aceE* | CGGCAAGATCATCAACGAACTG | TTCACGAACGTACGCACCATCT |
| *aceF* | CCGATATCACCGAGTTGGAAGC | CGCCACACCGATGTTGATGTAT |
| *aceK* | GGAAGTGAATTTCCGCGACATC | AGTAATCAGCGCGGAACAGGTC |
| *ackA* | TACGCCCTGCCTTACAACCTGT | CGGATAGCAGAAACGGAACCAC |
| *acnA* | CAAAGGCGATCCGGTTTATCTG | CTCCTGCCAACCGTAGGTATCG |
| *acnB* | GTGGATGATCGCGGAAGGTTAC | GGAGCACACAGGATTGGCTCTT |
| *acs* | GCGGCGCTGACCTTTAAATATG | CCAGTTGGGTACGCCTTCAAAC |
| *actP* | CTATATGTCCGCCGCCTCATTC | GTAAGAGGCCACATCGGCAAAG |
| *aegA* | CTTGCTCACTTTCGGCATTCCT | GAAGACCGCGTCGTATTGTTCC |
| *aer* | GACGATATCGCCGACAGTACCC | GCTGGCTAAATGACGCACTTCC |
| *agaA* | TGGAACTGATAGCCGATGGTCA | CACCGTGCATCTGCACTTCTTC |
| *agaV* | ATGGTACTGGCAGAAGGGATCG | TTGTTGTTTGCCATTGGCGTAG |
| *agaW* | TACGCTGGAACTGGTGTGGATG | TCTGTACTGCGACAGCGAAAGG |
| *agp* | CTCAGCAAACTCCAGCTTACCGA | TCTACCAGCGAGTTGCCGACTT |
| *aldA* | CAATTTGGTAACCCCGCTGAAC | ATCGACATTTCCTGGCGAACAT |
| *aldB* | GTTTACCGGCTCAACGGAAGTG | AGGCAAACAGTGCAAAGCCTTC |
| *ampD* | TGTTGTTAGAACAGGGGTGGCTG | AATAATGCGTCGATCCACGGA |
| *ampE* | GATGCCGTGCTTCATGTACTGG | CACCTTATCGACATGCGGTTCA |
| *ansB* | AATATCGGCTCCCAGGACATGA | GTTTGTCGCATTTCACCGTCAG |
| *araA* | TGTCGCCAGCTTGATTGATCTC | GTGCATGGCTGAAGACGGTATG |
| *araB* | CGATTGCAATTGGCCTCGATT | TGTAGTCACGCGGATGATGACG |
| *araC* | GGGATACTCGTTTAACGCCCATC | TAGTGATGAATCTCTCCTGGCGG |
| *araD* | CAAACGCCAGGTATTAGAAGCCA | CCATATCGTCAGCGGTCATGAC |
| *araE* | GTTGATGGTCACACTCGGCATC | ACGCAATACTTCTTCCGCCTCA |
| *araF* | TACCGCCAACGAACTGGATACC | CGGATGTTGAACCAGCATTGAG |
| *araG* | ATATGCAGCAGGTTGACCACGA | AACCGCCAGACTTATTGGCGTA |
| *araH* | CATTGGCAACGATGCAGATTGT | ATAATCAGACACGCGACGGTGA |
| *araJ* | CAGGTGTGTTTGCCTGGTTCAG | CGCAGTGGTGAATAACGTCCTG |
| *argG* | GATTGCCTGCGGTTTCGACTAC | ATCCCAGAATTTCACGCCCATA |
| *aroA* | CCACCGTGTTGATTGTTGTTGC | TGTGGTCGGCGGTTTGTAGTC |
| *ascB* | AACCAGTCTGAAGGTGCGTTCC | TGCCATCAGGGCGATATCTTCT |
| *ascF* | ACCGTTTCACAAGCCTTTCAGG | CATTGCCAGCAGTTTGACCATC |
| *aspA* | TGGCCAATATCGGTCTGGAACT | CACGCAGTTGGTTAATCGCATC |
| *bglB* | ATCCGGTTGGATTACGGGTTCT | TCGGCAATCGCTTCATTTACCT |
| *bglF* | TTAACAGTGTGGCAGGCCTTGA | CACTTTGTTCGGTCGTCCACTG |
| *bglG* | TTGCGATTAAACGCTTTCAGCA | TATTTCCGCTCATTTGGGCACT |
| *bhsA* | CGAAGTTCAGTCAACGCCAGAA | GCTGTTCCATGGAGGGTATTCG |
| *caiA* | CGAGGCTATTGGTCGTTTCCAG | TGCATTGCGCTATCCACAACTT |
| *caiB* | CCGATGAAGTACTGTGGCAGCA | GCTGGTCAACATCACCGTTCTG |
| *caiC* | ACAGATTCAGCAGGAAGATGCCA | AGAATTTCCGCCGTATCGTCAG |
| *caiD* | CATTACATCTGACCCGCAATGG | CTCTCCGGCACCGGTAATAATG |
| *caiE* | AGCGGGAGCCAATATTCAGGAT | AATGCTCTCTTCGCCAATGACC |
| *caiF* | AGCAAGGCGGTTAATACCCTGA | CTTCTGCTCGCGGTTAAGTTCC |
| *caiT* | TGGGTTGGGTGTTCATTGTCAG | CACAGTACGACCACGGGAGATG |
| *cdd* | GTGAGCGGAACCTGGTATTTCG | AACTGACGGCAGTGACCACAAG |
| *chbA* | ATCAGTCACGGATGGCATTGAA | TAATCAGTTCACGCGCAAGCAT |
| *chbB* | GTTCTGCGGGCATGTCTACCTC | ATCTGCGGCCCTAATAACACGA |
| *chbC* | AAGCAGCCACACGTTAATGCAA | TACGTTGCCGCCAATACCTTTC |
| *chbF* | GCACGAGCGCAGGTAGTACAGA | CCCATGATGCGGGATATTAACG |
| *chbG* | GCACTACCCAGGGATTCAGCAG | TCCGTTAAGCGTGGGAAACAGT |
| *chbR* | GCCTTATTGCTTTGTCGCTTCG | AGCCACTGCGGTACATCATCAA |
| *chpA* | AGGTAGCGAGCAAGCTGGACAT | TCAGCTAACGCTACGCCATCAC |
| *cirA* | TTTCTTGTGCATGGCCTGTGTT | TGTACGCCAGGCACTTCTTTGA |
| *cpdB* | GCCAGTTGACCTTCCGTAATGC | GACTGTGGTTTCGTGCTGTTGG |
| *crp* | CATTCTGATGCGTTTGTCTGCAC | CCGACAATCTGACCAATTTCCTG |
| *crr* | AATATCGAAGACGTGCCGGATG | TGTCGATACCGAAGTGGACGAA |
| *csgD* | TCCATGGAGGATCAAGAACGTG | GTTATTAGACGCGCCGATACGC |
| *csgE* | GAATCTTCACGCCGTTGAGGTAG | AGTGATCCAGCTTCCCCATCG |
| *csgF* | TTGGTATTGAAACACCCTCAGCG | CGATCTGTCACGTTCAACTGCAA |
| *csgG* | ACACGCAATACCAGCTCGATCA | CCGACATCAGGCACAGCATAAC |
| *csiD* | CGTCTGCTGGAACTCACCTTCA | GCAACGTCTTCAACAGCAATGG |
| *csiE* | TTGAAAGTGAATACGGCGTCCA | TTCGCGTAACTGCTGCTCAATC |
| *cstA* | CAGGGAAATACCTCGTCTGGACA | CACATAGTCCAGCCCGTCGTTAT |
| *cyaA* | GTGCAGGTTGAAACCAATCACG | TGACGTCACGTACCAGCTCCTC |
| *cyoA* | TGGAAGTACCGTGCGAGCAATA | CGATGGTAATGGGCTTCTCGTC |
| *cyoB* | ACTTGATGCAGTCCCGTTCCAT | AATCAGTACTGCACCGCTGGCT |
| *cyoC* | TGGCAACTGATACTTTGACGCAC | CACCAGAACGGCATAGGTAGCA |
| *cyoD* | CGTTCTGGATGGTGATGACAGG | GCGATGATTAGCACGGTGAAGA |
| *cyoE* | TGCTGGCTATCTTCAGCCTGTG | CGTAACCGCCAAGAGAGAGCAT |
| *cysG* | ATGCAGGCATGTTAACCCTCGT | CGCATCGACCACGTTACAGAAG |
| *cytR* | GCATGTTGTTGCTGGGTTCAAG | CAATCCGTTTATGCCCTTGCTC |
| *dadA* | GTTCTTACTCGACGGCGATGCT | CAACACGAATGCGGTTATCGAA |
| *dadX* | GCTTACGGGCATGGTATTGAGC | GTTGCTGTGTACGCAGGTGGTC |
| *dctA* | CGACGGCTTCGTTAAGCTCATT | AATCAGCGCGATGGTACTGACA |
| *dcuA* | GGTCTGTTCATTCTGCCGACCT | ATGAAGCTACCCAGCACGAAGC |
| *dcuB* | ATCGCTGTAGTCGCCCTTCTTG | CGATCATACCGGAACGGAAGAC |
| *dcuR* | CTGCATTACGGTGTCGTGGATT | AGGATCCTGTTCGTTGGAGCTG |
| *deoA* | CCGACCGACTTCGTTGAGAACT | CGACGCTGTAATCGATGGTGTC |
| *deoB* | TTCCGGTACGGTCATTCTGGAT | GATATTGTAGCCGCCGTTGGTC |
| *deoC* | GACGAGAAAGTGATCGCCCTGT | GCGATGTCGATGTCGTCGTTAC |
| *deoD* | ATCCCGTCCTGCTCCATCTACA | AGTCAGCGATAGCGGCAAAGTC |
| *dgsA* | ACCGGCGTTCCGGTTTATATTC | TGTGTGGCCTATTTCCACGAGA |
| *dksA* | AAGTCGATCGCACCGTTACACAT | GCCGAAATCTTCGTCTTCCACTT |
| *dsdA* | TCTGGGGTTATCAATCGGCATT | GACTGCGCTGCTTTACGTCCTT |
| *dsdX* | TTAGGCGCAACGTTATTCACCA | ATCGTCCCCATGCTCATATGCT |
| *dusB* | CCTCTTGCAGTACCCGGATGTC | TCAGAGCCTGAATGCCACAGTC |
| *ebgA* | ATGAGTGGCAAACCGACATCCT | CAGCACTTCACCATCGCTCTGT |
| *ebgC* | ATGCGCTATTTACCGGGCATC | GATTTGCCCTTCGTGGACCTC |
| *entA* | CGCGTATTGGCATGAGTGCTTA | ATACGCTGTTCTTCGGCGTCAT |
| *entB* | CTGCCGGAGTCTCACGATATTCC | GCAATATTCGCGATCACCTGC |
| *entC* | CCGCCATTGATAGTGGCGTATT | TCTGACGCCAGCAGACGATTAC |
| *entD* | CACTACGCACAACTGCAACACG | GCCAATCGGTTGACGAGATACC |
| *entE* | GCTGTCAGTTGCAGCAGGTGTT | TTGTAATAGCCGCGGAAGGTGT |
| *entH* | TGATCTGGAAACGCCATTTAACG | CCATGTAGTAAACCGAACGGCTG |
| *envZ* | GTTAAGCAACTGGCGGATGACC | CAATGATGGCGTTGCACTCTTC |
| *epd* | TACGGAATTGCCGTTGGTCTCT | CTAACGTCGTGTCGAGCATTCG |
| *exuT* | TCAGATCCTGCGTAACCGTCAG | ACCGAGGATACAACCGAGGTCA |
| *fadD* | TGACCGGACAGTATCTGCTGGA | CCGGTTGACCTGGTGGTACTTC |
| *fadH* | CGGCATTGCACCAGATTTAACA | ACCAGATGCGGTTGGTAGCTGT |
| *fadL* | CCGATGATGCAGGTAACGTCAG | ATCGGTGCAACAAAGTGCATGT |
| *fbaA* | ACTGTACACCCAGCCGGAAGAC | TTCCTGAGAATCACGCAGGATG |
| *feaR* | GGCGCTGGCGATATTACGTTAC | ACAGGCGATGACTGAGTTGCAC |
| *fecA* | GCCAGCTATGCGTATGTGAACG | GTTATCCGCAAACTGGCTGGAC |
| *fecB* | CCCACTATCGCGAAGAGAGCAT | TAAGCGGCTGATGGTGGAAGAT |
| *fecC* | TTCACTGCTCGGCATTAACAGC | GTATGACGAAATCCGCCTCCTG |
| *fecD* | TGCTGAAGATGCTGGCAAAGAC | TGGCAATCTTCACAAAGCTCCA |
| *fecE* | GGTCGTAATCCCTGGCTGTCAC | GTATTCTGGGCCAGGACCATTG |
| *fepA* | AACCTGACCGGTAACTCCACCA | GGCACCCAGGAAGTATCACCAC |
| *fis* | ACGCGTAAATTCTGACGTACTGACC | GGCTGTTCTACTTCAGCCAGTACCA |
| *fiu* | GCGTGGTGCCGATACCTCTAAC | CGGCTGCTTGCTGATCATATTG |
| *fixA* | ACAGCCTTAAGTGTGGGCGGTA | GGGCATAAAGGTCGGAAGAACC |
| *fixB* | AACAGCTTTGCAAGGCGATAGG | ATTGATGGCGAAAATGGTTTGC |
| *fixC* | ACTGGCGGAATATCGTCAGCAT | GGTCACTCCTTTCATGCCATCC |
| *flhC* | ATGCTGCCATTCTCAACCGACT | ATGCCAGCAGTGGTCCTTCTTC |
| *flhD* | AGCGTCCGCTATGTTTCGTCTC | TGATGCCGGTATGAATTTGCTG |
| *focA* | TGTTGATTGTTGTTGCTAAGGCG | TTTGTGGTCGGCGGTTTGTAG |
| *focB* | CCCGTCTTCACAGGCATTAACC | GGCAATACCTGCCAGATTACCG |
| *fucA* | TTGGAACACGCGAACTTTCTGA | TAAAGTTGCGCCAGCACTTCAA |
| *fucI* | GATGCCGATGACACATCGATTC | CATATCCACCGCCTGGACTTTC |
| *fucK* | GCCCAGGTTGATACTTCGCTGT | CAACATTTGCCAGGGTGTTTCA |
| *fucO* | GGGCGTGAAAGTGGAAGGTATG | CCACCGGTACAAACATCATCCA |
| *fucP* | TTGCAGCCGGATTAGGTTGTCT | GAACGTCTTGCGATTGATGTGG |
| *fucR* | CGATAGTCGACCTGCTGCTGAA | ATGTGAAAGGGATCGCCACTGT |
| *fucU* | CCAGGCGATTATCCCGTTATTTG | AAACGATTGATGCGGATGATGTC |
| *fumA* | CGATGAAAGAGATCCTCGCACA | ACCCGCGTAGTAAATCGGATGA |
| *fumB* | CTGCCGGTTATCCATCAGGTTC | GGTAGAAGCCGCCGTGTTTATG |
| *fur* | AAGAAAGCTGGCCTGAAAGTAACG | CAGCGTCGTCAAACTGGTTCAG |
| *gabD* | GCGCAACTGGTTCAATTTGATG | CTGATGACCAGGAATGGTGTCG |
| *gabP* | TATTACGCGCCTGCGAAGGTAT | AAAGCCGATGACTAGCCAGGTG |
| *gabT* | TCGCCATTGAGCTGTTTGAAGA | TCAATGGTGAGCGGTACAAGGA |
| *gadA* | CTCTCTGAACGTCTGCGTCTGC | CTGGGCAATACCCTGCAGTTTC |
| *gadB* | GCTTATCTGGCGGATGAAATCG | GCAGACGCAGACGTTCAGAGAG |
| *gadC* | ACTGCTGTTGCTGGGTGTTCTG | CGTAATCACCAGCTGCGAAATG |
| *gadE* | TGGTAAACACTTGCCCCATAAGAA | CAGGGTGACGATGTCGCTCATA |
| *gadX* | ATGCGAACACGAGTTTGTACGG | GTTGCAAAGCACGTTGCATTCT |
| *galE* | GCTGGACGTGGTTAATGCCTTC | ATATCCCTGTGGATGGCGTGAC |
| *galK* | GTCGGAACCGTATTGCAGCAG | GCAATCGATCAGCAAGGCATG |
| *galM* | ATGCCAACCGTATCGCCAATAG | CGTCGGTCAGACGATATTGCAC |
| *galP* | CCGGGATGAACGTCATCATGTA | CCCAGCGTTAGCGTTGGTTTAC |
| *galS* | ATAACAGCACGCTGGTCAGTGC | AACGCATCAGAAACGTCCATCA |
| *galT* | CAGAGCTCAGCGTTGCAGCAT | GCTATTTGCCCAAATCTGACCGT |
| *gapA* | ATGAAAGGCGTTCTGGGCTACA | GGAGTAACCGGTTTCGTTGTCG |
| *gatA* | GGCGATTCATGCTAAGTCGTCA | TCGACGATATCGGGCTGTTGTA |
| *gatB* | AACGCAAGATTATTGTCGCTTGC | TCCACTTTGGCAGTGGTGCATA |
| *gatC* | CGCTAAGCAGGCTCGTAGTCGT | TGATTACCCGGCACACAAACAG |
| *gatD* | TTGAGCCGATTACCGTTGGTCT | CTAATGTCGATCGCCGTCACAC |
| *gatY* | GTGCAAGTCAATGAAGCCGATG | AGTTCACCCACTGGCGAATGTT |
| *gatZ* | ATATGTTCGTGCCGGCTTCAGT | CGGTGCCAATGACATAGCTCAG |
| *gcd* | GGCAATTAACAATACAGGCTCGC | AGCATCACAAGGCCAGCGATA |
| *gdhA* | CTCGTGTGATCACTGCGTCAGA | GGCTGTTGGCCTTCGAGATAGA |
| *glcC* | TCGCCATTTGTCAGGCTTCTC | GCAACACCGCGTTGTAGATCC |
| *glgA* | GTTCAGAGATGTTCCCGCTGCT | GACGGGATACTACCTGCGCATC |
| *glgC* | ACTCTGGTGCAGCACATTCAGC | TAACGGCGGATAATGTCGAGGT |
| *glgP* | AATCGGCAGCGGTGTATTCAGT | CCCATATTGGCAATGTTCAGCA |
| *glnA* | AGATGGGTCTGGTGGTTGAAGC | GGAGCCGTTATCACCGAACATC |
| *glnG* | TAGTTCCATCCGTTGGGTGCTT | TGGCGCTGTTTAATCTGCTTGA |
| *glnL* | ATAACCAGCGCCGCTTAAGTCA | GGTCCGCCTGTTCGATAATCAC |
| *glpA* | CACCGTTGATCCATTTCGTCTG | GTTTCGCCGGTGAGATGGTTAC |
| *glpB* | CCAGTTACAGCGCCAGTTTGTG | CGTTACGTTCCGCTACCAGTCC |
| *glpC* | TTAACGGTGCTGGATTCCCAGT | TTACAGGTTTCGCAGTCGGTGA |
| *glpD* | AGCGGAGCTGAAATACCTGGTG | CAGCGATAACCTCTGCTGCGTA |
| *glpE* | AGAGTTTCGCAATGGGACATGC | CGTAGCCCTGTTGCAGCAGATA |
| *glpF* | GGCTCCCTTGCTGATTGGTCTA | CGGCACCAGGAAGTAAGGAATG |
| *glpG* | ACCCACGCGTTAATGCACTTCT | TTGCTGCACATAGCCGCTTAAC |
| *glpK* | ATACCCTGGACTGGGACGACAA | TTCACGCACAACTGACCAAACA |
| *glpQ* | GACTGGAATGAAACGCAGCAGA | GCTGCGATGTCTCCTCAATCAA |
| *glpR* | GCGCATTGTCACCAACAATCTC | GTCGCTATCGATGCCGCTTATC |
| *glpT* | ACACTGGTGACCATCGCGACTA | CAGGTAACCAAACAGCCCGGTA |
| *glpX* | AGGCGATAAAGGCTGCTTCCTC | GTGGCGTGGTTTAGCCAGAATC |
| *gltA* | GATCAGCTGGCGACCGATTCT | ACATGACTGCCATTGGATGCG |
| *gltB* | CACCAGCGCTTCTCCACTAACA | AGTCAGAGCCGGTTTCGTTGAC |
| *gltD* | AGCCGCTGGGTATTGAAGTGAA | TCCATGTTGTGTGGACGGAAAC |
| *gltF* | CGCTGCATCCATTGGTATTGAC | AGCAACTTGTCAGCGAGCAGAA |
| *gntK* | CTGAATGACGACGATCGCAAAC | CTTTCAGGCGGCTTTCAATCAC |
| *gntP* | CAGGTCTGATCCTGCCGAAGTT | TTGGCGATGGTGGTAGAGATCA |
| *gntT* | TGCTGATTGCGATGTTCACCTT | ATGTTGGTTTCGTGCATCATGG |
| *gntU* | TTCGATTGTTGTCAGCCACGTT | CAACAGCTGGAACGCAATCATC |
| *gntX* | ATGCCACTGGCGTTAGGTCATT | ATAGTCGGCAACCGTGACCAGT |
| *grpE* | AAATTCGCGCTGGAGAAATTCA | CGCCAAACTTACGCACAACATC |
| *guaA* | AGAACTGCGTAAAGCGGACCTG | CCAGTGTGCGGTCATAAAGTCG |
| *guaB* | GAAGGCGGTATCGGCTTTATCC | AACCGTTACGCTCGGTCAGTTC |
| *gutM* | GCATCGTCGACACCTTGTTTATGA | CGTTTCAGTTTAAGCGCCAATGAT |
| *gutQ* | GCGCTCGCTTGCTGAATAAAGT | CGTAAATCGCCGTCGGTAAAGA |
| *gyrA* | AGATGTCCGAGATGGCCTGAAG | ATGCGGACGATCGTGTCATAGA |
| *hlyE* | CAGGAGTATTCACAGGCAGCCTC | GGGCGGATGCTTTCTTCTCAT |
| *hofB* | GAATATTCCACAGCTCACTGCCC | GCGTCCAGCAGGTGATCTCAA |
| *hofC* | ATGGCGAGTAAGCAACTCTGGC | CAGAATTGATGGCGATTCGCTT |
| *hofM* | GCAGCGTCATCACATTATGTTGG | CACGTTATAGGCGGGACTGAGTG |
| *hofN* | TTTCTGCGTTTCTGGTTGCTGA | TATCTGTAAGCTGCGGGCGAGT |
| *hofO* | TGGTAGACACCGCTCCCTTCA | CTCTGCCAGCCGTGTAAATGC |
| *hofP* | CGAGCGCATCATCGGTGTAAT | TGCGGCTATCCATTGCTTCAT |
| *hpt* | ATATGGTGCTGGTGGGTCTGCT | CGTCCTTGCCACGGATATCTTC |
| *hupB* | TGGCCGTGCGTTAGATGCTAT | CTGCACGGAAGCTCGGTACTTT |
| *hyfA* | GGTGAAATGCGACCTGTGTGAT | TTGCTCCTGAGTGAGGGAAAGG |
| *hyfB* | GATGATCGTCGCCATGCTACTG | ATGAAACGTGCCGGGTACAAGT |
| *hyfC* | CTTGTCGTTACGCTGCTCAAGG | CGGTTAACCAGAAGACCCATGC |
| *hyfD* | TCATGGCACTGGTCACCATTCT | GAACGCGTGGTTGACGATGTAG |
| *hyfE* | TTATTCGGTCCGGCAATGATG | CAGGATATTGCGCTGGCTGAC |
| *hyfF* | AAGCAATCGTCCTTGCTTGACC | AGTTCAGCAATACGGCGGAGAG |
| *hyfG* | GATGACTGGCCGGAAGATATGC | CATCCACAAACAAGCGGAAGTG |
| *hyfH* | TGACTATCCAGACCGACGACCA | CGCGGGTATAGAGATCGGCTTT |
| *hyfI* | ATCACAGCGTATCGCCATTGAG | TACGCGAAAGAGTTGCTGAACG |
| *hyfJ* | ATCACGTTGGCGTGATTGATTG | GTGTGGCTGGCGTCAATAACAA |
| *hyfR* | GAAATTGGCGATCTGCCGTTAG | AAAGAGATCGCTGCGAAACTGG |
| *idnD* | AACGACGACATGGATCACTGGA | ATCATTGGGAATTCTGCCATCG |
| *idnK* | AGCCCCCATGTTCATTTCCTCT | CGACATTGTTCGGTGACATTGG |
| *idnO* | AAGCACTGGTTGAGGACGAAGC | TAACATGCCGCCATCAACAAAC |
| *idnR* | ATTACGATCGCGATTCCGAAGA | CAGCCGTTCTCCCTGTACATCC |
| *idnT* | GCCATCGCGACTATCTTTGAGG | TGTTCCAGAAGGAGGGCATCTC |
| *ilvB* | GGAAGTGGACACCTACGGCATC | AAACTGCCGTTTGCACATCCTT |
| *ilvN* | CGTAATTCTGGAGCTCACCGTTC | CTGGTCGTCATTGACCAGTAGCC |
| *infB* | GCGGTTAAAGGCTCTCAGGTCA | TCATTACCGCCTCTTCCAGCTC |
| *kbaZ* | CGGCGATTGAAGAAGAACTGGT | CTGGCTGTCCGGCCAGTAATAG |
| *lacA* | CCGATATGTGCGAAGGCTTACC | GCGGCCTATATGGATGTTGGAA |
| *lacI* | CTGTGGAAGCTGCCTGCACTAA | GCGCCGAGACAGAACTTAATGG |
| *lacY* | TTAGTGATGTTTGCGCCGTTCT | GAACATGATGCCGACAATCGAG |
| *lacZ* | ACCATGATTACGGATTCACTGGC | ATTCAGGCTGCGCAACTGTTG |
| *lamB* | CTCCACCATCTGGGCAGGTAAG | TTGCTGGCGAAAGAGGAAGAAC |
| *lhgO* | AAACGGCATTCGCTACGACAA | GAGCCCGGTGATATTCGGTTC |
| *lpd* | GGTGTTCACGAAGGTCACGTTG | AAGCGATAGCACGACCAGAAGC |
| *lsrA* | GAGAACGTTTACTGCGCGGTCT | TTAATATTCAGCGCCCGACGAT |
| *lsrB* | TCGCCTTACTTAGCGCTCTTGG | CATCAACGCCCAGCTCTTTACC |
| *lsrC* | GTTTATAGCAGCGCGCAAATCC | AAAGTCGCGACACAAGCAACAG |
| *lsrD* | TGGTGGTTCCGGTTCCATTATC | CGCCAGCCACTCTTTAATTTGC |
| *lsrF* | CAAAGATATGGTGCGCGATCAG | GATAGCCTGCCAGCACATTTCC |
| *lsrG* | TGCGCTTCGATGTCTTACAGGA | CGGCATCAAACCATTGAACAGA |
| *lsrK* | TTCGGGCTGTGATATTCGACCT | CGAAACGGCAGCGATATACTCC |
| *lsrR* | CAGGACGATGCGACAATCATTC | GCGCGCTTAAAGGTAAGCCAAT |
| *lyxK* | GGGGATCGCTGAAATCAATCAC | CCGCATTGAGGGTAAATTCGTC |
| *malE* | CGTACGTTACAACGGCAAGCTG | GCGCTCTTACCTTTCGCTTTCA |
| *malF* | GCACAAGGGGAATACCTGTTCG | TGCAGACCAGAGGGAAGAGGAC |
| *malG* | AGTACCGATTCTGGCGGTGGTA | GGCAAAGTCACCCCACAGGTAG |
| *malI* | GAATCAGGGTGTCGATGGTGTG | GATGCTCCGTCAACAACTGTGC |
| *malK* | GCGTATCGAAATCTCCCGTCTG | AACGGTCTGCCGGATAGTGGTA |
| *malM* | GAACATGACCCCATCAGCCTTC | AGTTGGGTCGTCTGCTGGAGAT |
| *malS* | ACGGCGTGATGCAAAGTGACTA | CATGCGACGAGAGGTAGCTCAA |
| *malT* | CTTAATACGCTGCCGGAACTGG | CAGTACCTGCCATTCACGTTGC |
| *malX* | GTCGGCCTGGTGATTCCATTAG | CCACCAGAATGTGATGCAGACC |
| *malY* | GGTGGTGTATGGCCCTTCTGTC | ACCAACCATCAGCCTGCTTCTC |
| *manX* | AAGGCGTGAAAGCACTGAAAGC | CGGGAGACATTGGTTTCTTTGG |
| *manY* | CAGAGCATTGGTGCAGGTATCG | GCATTGCTTGCAGGAACAGAGA |
| *manZ* | ACTGACCAGACGGGCAAAGAAC | ACGAAGAAGCCAACGATGATCC |
| *marA* | GGGTTACTCCAAATGGCACCTG | GGTTCGGGTCAGAGTTTGTTGC |
| *marR* | CTGGATATTACCGCGGCACAGT | TTCCACCCAGCCTTTACAGACC |
| *mdh* | CAACACCTTTGTTGCGGAACTG | GTTCTGGATGCGTTTGGTCAGA |
| *mdtE* | TTCAGGGCAGTACGCCAGTACA | CAGCAAGTCACCATTTGGGTTG |
| *mdtF* | GTGCTGGCGATAGGGTTACTGG | GCGGAAAGAACAACGGCAATAC |
| *melA* | ACCCAATGGCGATGAATACCTG | TATGGTTGATACCTGCGCAACG |
| *melB* | CGTCTGCTGAAGGAAGCCATCT | CAAATCCGCATCACCGATAACA |
| *melR* | TGCCGATGCATCTGTTTCTCTC | CAATTTCATCAATGGCGAGCTG |
| *metK* | AGCGCTATCGGCAAACAGTCTC | TCAGCCTGACGCTGTACCAGAC |
| *mglA* | GTTAATTGCCGAACTGGCGAAG | ACGCAAGACGCAGAATTTCGTT |
| *mglB* | GCGCTGAAAGCACACAACAAGT | ACCTTTACCATCGGCCAGGTTT |
| *mglC* | GGCCTGAACCTGCTGATGATCT | GCTGAACGATACACCGCCTACC |
| *mhpA* | CCGTATTGATCGCGTACTGCTG | CGTCACGGACAGGTCAATCATC |
| *mhpB* | CAATAAACGCGTGCTGTTCCTG | ACTTCTCAGCGGCGCTAATCAC |
| *mhpC* | GCCTGGAAGCTAATCCGAAACAG | CACAGTCGCGGAAGATATGCAG |
| *mhpD* | AGCATACTCTTGAGCAACTGGCG | TTGCACTTTCGGATGTGTCAGG |
| *mhpE* | CGCCATTCGTCATCAGTATTCG | CCGCTTCAATCCATTCAAGGTC |
| *mhpF* | AGCGTAAAGTCGCCATTATCGGT | GCATGTTCATCAGTCCGATCACC |
| *mhpR* | CACCGTTTCAGCCGCTTATCCT | CGCGGTAGTTCTGTCCGTAACC |
| *modA* | AGAGAAAGGCGTGGATGTGGTTT | TGTCTGACGCGTAGCTGTATCGA |
| *modB* | GGTTACTGGTGCGTTGCACGT | CCATCAGCGGAAACGACATGAC |
| *modC* | CTTATCTGCAACGGCTGACACG | CCACGGATTCATCACGCTACTG |
| *mpl* | CGAATGGCGTCACGGTATATGA | GAAGACTTCATCGGCACGACCT |
| *mtlA* | TCGGGCCGATGATCACTTATCT | GCGGACCTGCAATCATAGAACC |
| *mtlD* | CGGTCATCAGACCATTCGTGAC | GGCCTACGCGCTCTACATCATC |
| *mtlR* | CGAGGCGGTAAATCTTCTGGTG | TCAGCAGTTCCGCATCTTCGTA |
| *murP* | GGTACAGGATGCCGCAGAAATC | ATTAACGTCGCTATCCCCAGCA |
| *murQ* | CACCGCTGAGTTTGCCATTACA | CATCAGGTTGCCGAACACTTTG |
| *nadC* | CTCGCCGCTATAACCCTGACAC | ACACCTCTTCAACCCAGCGTTT |
| *nagA* | ATGTATGCATTAACCCAGGGCC | CAGCCGTTTAACTGCACATCGAT |
| *nagB* | TCGCCATATCGTCAATCGTATCA | TTCGTCCATGTTGAAGGTGACAA |
| *nagC* | ACCAGGCGGACAAGCTCAGAT | CGTTCGATAAGCTGACGCGTAA |
| *nagD* | ATGTAATTTGCGATATCGACGGC | ACACGCTGTCAGGTACATCGACA |
| *nagE* | GCTGGTGATGGGCGTTATCTTC | GTAAGGCGCAGACGGGTGATAC |
| *nanA* | CCGTCACGCCGTTCTACTATCC | ATCGCCAGAGGTCTGTTTCAGC |
| *nanC* | GCATGGCAAACGACGCTATACA | TCTGCCGTTGTAAACACCCTGA |
| *nanE* | AGCGTGCCGATTATTGGAATTG | GATGGTGAATACGTGCCAGCAG |
| *nanK* | AGAAGCCTTGCGTGATGCCTTAT | CGGCAAATTGGTAAGTTGTTCCA |
| *nanM* | AAGCTTGCTCCCGTCTCATCAC | CCATTTCCCGTTATGCCAAAGA |
| *nanT* | TACCTACAACGTTGGCGCATTG | CGTCATGAGTACGCAACGCTTC |
| *narQ* | GGATACCAACCTGCCGAATGAG | CGCCTGCGAACACTGATACAAC |
| *nfuA* | CGTATTTCCGATGCTGCACAAG | CTCATCAACATACGCGGTCAGC |
| *nirB* | GGCAAAGGCTACGGTTGTGAAG | GATCACCGAGTAGGTGCCGTCT |
| *nirC* | CGGTAATTTGCTCGATCCATCC | ACCAGGTTACCCAGCCAGGTTT |
| *nirD* | ACATCGACCCGTTCTTCGAGTC | CACTCGCGCTTCGTAATGTTTG |
| *nlpE* | CTCGGTGCTCGTGAAGAACCTT | AGGGTCATCGGCGTCATAGGTA |
| *nrdA* | TCAGTCGATCTCTGCCAACACC | CACCAGATCGTCTTGTGCGTCT |
| *nrdB* | GATCCTGAGATGGCGGAAATTG | TGCCTGCATACGGATATTGGTG |
| *nupC* | GCACTGTTTGCCACCGTTACTG | CAGATCCATCATCGCAACGAAC |
| *nupG* | TCGGCTTTATCATGGCAATGTG | AGGGTTGTCCAGCTCTGATTCG |
| *nusA* | TTGAAGCCGCACGTTATGAAGA | CGGTGATGATTTCACCTTCGTG |
| *ompA* | ACTCGTCTGGGTGGCATGGTAT | GTCACCGATGTTGTTGGTCCAC |
| *ompF* | CGCGGGTCTTAAATACGCTGAC | AGTTCAGGCCATCAACCAGACC |
| *ompR* | CGCCTGCTGACTCGTGAATCT | TAGTCGTCAGCGCCAATCTCC |
| *osmY* | AGAAGGCTCGGTGAAGGGCTAC | TTGTGCCTGAGAATCGACGGTA |
| *oxyR* | TATGCTGCACCAGACCTTTCCA | TAGCCAGCAACATTGGCTCATC |
| *paaA* | AACGCTTTGAGCAACGGATAGC | CTGCACTTTGGCCAACAGAATG |
| *paaB* | GGCAAACAGGGCTTATCACACC | TTGCGAGGCAACAATTTCACTC |
| *paaC* | TCAGGCACGCAACTTCTTATCG | GAAATACTGGCGTGCAATGGTG |
| *paaD* | GATCCCGGTGCTGACCATTACT | GAGTTGCAGCACAACCTGAACG |
| *paaE* | GCAATTGCAGCAGGATCAGGTA | TGTCTTTCAGGTCTGCCAGTGC |
| *paaF* | ACCAGCATTTCGGTCTGTGTGA | ATTGACGGCTGCGATAAGTGGT |
| *paaG* | TAATACCCGATTGCGGTGGAAC | CTGCCAGCGTTTCATCATCAAC |
| *paaH* | TTTGCTGTCACCTGTTCGGTGT | AACTGTCGCTTACCGCTTCCAG |
| *paaI* | GCCCATGCAATGTATGAGAACG | CTATTGCAGGCGTAGGCAAAGG |
| *paaJ* | GCTGCGTTGATTATTGCCAGTG | CGCTTCGTTCAGTTCAATCACG |
| *paaK* | CTGAAATGGACGCTCAAACACG | GGATAGTTGTCGCGCAGATCCT |
| *paaZ* | AGGAGGCGGTGAAGAATTAGGC | CTGTCGCCTGGTTGTAGCTCCT |
| *pdhR* | ATTCAACGTCTCGAAGCGAAGG | AGTTCACGGATGCGTTCCTTGT |
| *pflB* | ACGACATCTCTGGTCCGGCTAC | GCGCTTCTTGTTCGGTGATCTT |
| *pgk* | CCTGGATATCGGTGATGCTTCC | TCGCTGTCTGCGATAGCGTTAG |
| *pncB* | ATGCAGCAAGCCGTGTTTCATC | CGTTGGACACGGTGACTTGTTC |
| *pnp* | GGCCGCGTCTACACTGGTAAAG | CACCGGTACTTCCTGACCCATC |
| *poxB* | CAGCAAGGTGGATATGGCACTG | TATTGCGGGTGAATGGCTTTCT |
| *ppdD* | ATGGTGGTTATTGGCATCATTGC | CACCGTCGCAGGTATCTAATCCA |
| *ppiA* | CGCCACCAGCCAGTTCTTTATC | CACATTCTGGTACGGACCAACG |
| *preA* | ATGTGTGCCAAAGCCTACGACA | CCAACGGATGTTCAGCAATCTG |
| *preT* | GTCACGACGCTCCCTGTAGTCA | TAATTTCTCCGTCGGGCACACT |
| *proP* | ACCGTCCTACGACACGATTGGT | AACCCGGCAATAGAACCGAAGT |
| *prpB* | CATTGCAGATTGTTGGCACCAT | GTCGGTCAGCACATCATCAAGG |
| *prpC* | ACCGCGGCTACGATATTCTTGA | GTAAGGCTTCCAGCACGGTACG |
| *prpD* | CTTAACGGCGGCAGATTACGAG | GACCAATGGGGTACTCCACCAC |
| *prpE* | CAATGTTGCGTTCATTGGGTGT | ATCAGCACCGGTTTAGCGTCAT |
| *prpR* | ACTGCGCAATATGATGGAACGA | CCTAAATAATTCGCCGCTGCTG |
| *psiE* | CCGTACTCAATCTTGGCCTGCT | CACAATCAGCGCGATAAATTCG |
| *pta* | TGCTGAACGTGGTATCGCAACT | GGTTTCGGTCATGCCTTTGTTC |
| *ptsG* | TACTGGCGTTGTGCTGTCCTTC | CCAGATGTGGTGCAGACCAAAC |
| *ptsH* | GCCCTGCTGCCCAGTTTGTA | TTCAACCGCTTTCTGCTCGTC |
| *ptsI* | AAATTTCTGCCGACCAGGTTGA | CCTGCTCCAGCTCCTCATCTTC |
| *putP* | TCACCATTTATTGCGCTTCGG | AGGCTGGCCTGTACAGTGTCAGT |
| *rbfA* | GGTTAAAGCGGGCATCAAAGC | CCGGGTTAACACGACGTTCTTC |
| *rbsA* | GGCATGAAGTCGTTACCCGTTC | ACTCACAGCCTGCTGTTCATCG |
| *rbsB* | TGCCAAAGTTATCGAGCTGCAA | GGATGAGCGGTCAACAGGTTCT |
| *rbsC* | GGCTGGTTTGGTATTGGTCGTC | TGATACCAGAAAGACGCGTTGC |
| *rbsD* | ATCGCGGAAGAGATCAAACACC | ACATTCTCCGCTGCGAATTACC |
| *rbsK* | TATGGGCTAGCGTGAATGGTGA | AGGTTGTGCGCCTTTACGTGTT |
| *rbsR* | CGCGTAGCCTCAAACTCAATCA | AGATTGCGATTCATCCGCTGTT |
| *relA* | GAAACCTGGGCGTATTGTCTGC | ACCGACTTACCGACGCTCTCAC |
| *rhaA* | GGTGCATATCGGCCTTGACTTC | ACGGCAACGATTTCTGCTCTTC |
| *rhaB* | GCCATTCGCCTTGGATTAAACA | GTTTGCCGAGTTGTTGTTGTGC |
| *rhaD* | CCGATATCGCACCATATCACGA | GATGCCTAAGTTAGCCGCAGGA |
| *rhaR* | ACCACACTGGCGCTTAGGTAGC | TGGTGTAACGATGGCGATTCAG |
| *rhaS* | GGATGGGCAGTATCCGTCTCAC | CCAGGTTCTCCTGCAAACTGCT |
| *rhaT* | CCATCAGCGCCCTGTTACTACC | CGTCATCAGCGTACCGACAATC |
| *rimP* | CCACCAGGTAAGTGCTGTGCTG | GTACCGCCATACGGAGAACCAG |
| *rof* | TCTTGAGCTGAAAGATGGCGAAA | GGATTCGCTTACCACCACCGTA |
| *rpoH* | CCGTTCACTGGATCAAAGCAGA | CTTTGCTGGTTACGCCCAGTTC |
| *rpoS* | GTTGATGACGTCAGCCGTATGC | CTCGAACAGCCATTTGACGATG |
| *rpsO* | GGTAGCACTGCTGACTGCACAGA | TCGATGAGCTGGGTGTAACGTG |
| *rrsG* | GGTGTAGCGGTGAAATGCGTAG | TCAAGGGCACAACCTCCAAGTC |
| *sbmC* | CGGTAGCTCGTGTAGTCGGTGA | GCTGCACCGCAACATACATTTC |
| *sdhA* | GTGGTGGGTTGTACCGCACTGT | TCCATATCCTGCACCGGTACG |
| *sdhB* | ATGCTGCTGGATGCGCTTATC | AATCTTCTTGCCCGGCTGGTT |
| *sdhC* | GCGATAGCGTCCATTCTCCATC | GTGATACGCCAGAGCGGTAAGG |
| *sdhD* | TAAGCAACGCCTCCGCATTAG | GGCGAAGAAACCGATCCAGAC |
| *serA* | ATTGGTACGCAATTGGGCATTC | TTTGGTGGACGGATTCTCTGGT |
| *serC* | CATTCTTTCCCGTCCGATTGAC | GAGCCGTTATCGTTGAGGATGG |
| *sfsA* | CGCTTTGGGCTAACAGGTTGAC | TCCCTGTTCGTTCTCCGCTAAC |
| *sgbE* | CTTCGAAGAACGTGGCAGGAGT | CTGGCGCGAGAATAGACCCATA |
| *sgbH* | GTTCGGTAACTGGACGCTGGAC | CCGCCAGTAATGGAAAGCTCAA |
| *sgbU* | AAACTGGGCATTGACCGTATCG | TCGGTCCACATCTCAATCAGGA |
| *sodA* | GAAAGCGGCTATCGAACGTGAC | TCAGCGGAGAATCCTGGTTAGC |
| *sodB* | ACTTTGGTTCTGGCTGGACCTG | CAGATAGCCAGGACGTGCATTG |
| *sohB* | AGGTTGTGCTCCGTCTGGAAAG | GCCACACAGGCCATCATGTAAC |
| *speC* | CGCCGATATGTGTAACGCTGAC | TAACAGCGCATTCGTCACCACT |
| *srlA* | CCGCTGTTGATTAGCCTGCTG | GGCTTAGGGTCATCGGATTGC |
| *srlB* | CAGTGACCAGATGCTCATCACCTT | CAGTTCGCGAAGGTTGTCTTCC |
| *srlD* | CAAAGTGGGCAGCAAACACAAC | TGTGGCAACAGTGACTGGAACA |
| *srlE* | CTGGCGCAGTACATTGTGGAAG | CCACCTTCGCCAGTAAACCATC |
| *srlR* | CTTTGACACCACAGGCACAACC | CTAACGGCAGCTTCTGCAATCA |
| *sucA* | CGATTGGTGCCGAGTATATGCA | GCGCCGAGGTAACGTTCAAGA |
| *sucB* | TCTACGTGAAAGCGGTGGTTGA | TTCTTCTCGATGTCTGCCATGC |
| *sucC* | CCGGCTAACTTCCTTGACGTTG | GTTCGGCGTTGTTACCTTCCAG |
| *sucD* | CGGATTACGGTTTCGGTCAGTC | CCACAACTGGCTTGGTAACGTG |
| *tam* | GTCGATATCTGGCGAACCACCT | TCTGTCCGTTCTCTTGCAGTGG |
| *tdcA* | GTTCCGAATCCATTACCCGTGA | GAACGAAGACAGTTGCGCTTCA |
| *tdcB* | GCGAAATTGTCGAAATGGAAGG | CAATTAAACCGCCACCACCAAT |
| *tdcC* | CATCATCGCACTCGTGGCTATC | GTAGGCAACAACCCAGGTGGAG |
| *tdcD* | TATTCACCGGCGGAATAGGAGA | CGGAATAACGGCACAAATGACA |
| *tdcE* | TCTAACGTGGTGTACGGCCAGA | ATCCCATCTTTGGCGTAGGTGA |
| *tdcF* | GCGTTGATTTAGGCAGCATGG | GATGATATCGCCCACGCTCAG |
| *tdcG* | TGACGGTATTGAACGTGGGATG | GCGCGTACATGTTGATCCAGTC |
| *tnaA* | TGCTGTGCATGAAAGACGACAG | CGATAAGCCAGCCAGTCGAGAT |
| *tnaB* | CATTGTTCCTCGGGCTGAAGAT | GGAATATTGCCGTGGAAACCAA |
| *treB* | GTTCTGGTCTGGCAGGATTGCT | CAGGCGGTATTTCCGCTGATAG |
| *treC* | AATGTCCTCCACCAGCCTTGAG | TTGTGCATTCCTTGTTGCCAGT |
| *trg* | GGCGATCTGACGATGAATGATG | CTTGTTCTTCGGTGCGAGATGA |
| *truB* | GTTATTTACCTGCGCCGTCTGG | CGGAAGATTCACCACCGGATAG |
| *trxA* | TCTGGATGAAATCGCTGACGAA | CCAGGTTAGCGTCGAGGAACTC |
| *tsx* | TGACCTTAGCTTCGGTCCGTTC | CGCATAGACGTTCATGGACAGG |
| *ubiG* | TTCAGGTGGATTACGTGCAGGA | ACTTGCCGTTGCGGTTAAGTGT |
| *udp* | GAATGTACGACTGCGCTGGTTG | ATCGCCTGCCACTCTTCCATAG |
| *ugpA* | CGGTGAGTTTCTTCCTGCTGGT | GAAGCCAGATCCAGTCCGGTAA |
| *ugpB* | GTTCAATAAGCCGGAGCAGGTG | ATTTGGCGTACTCGCGAATGTT |
| *ugpC* | TGAGCCGCTCTCTAACCTCGAT | ACCGCCGTTCATCACCATTACT |
| *ugpE* | GATCCTCTTCCCGCTGTACGTG | TGTTAAGCAACATCCGCCAGAA |
| *ugpQ* | GCTGGGCTGCGTCTCTATTCAT | GACCAATCACGTCAATCGCATC |
| *uhpT* | AACATCGCGCAGAACGATATGA | AGCATGAACGGCAGGAATTGTT |
| *uidA* | TATTGCCAACGAACCGGATACC | ATAACGGTTCAGGCACAGCACA |
| *uidB* | GCCGCTTCATTGACCTTTGTCT | ACATTCTCACGCGTCGATTTGA |
| *uidC* | GTTATTTGCGCCATGCAATGAA | TCCGCACATCTCGTTTACTGGA |
| *ulaA* | CCGTTGTTGCTGGGTATTGTGA | CGCCGTTAATGCCGTAGACTTC |
| *ulaB* | ACGGACAAGGCAGTTCCATGAT | GCAATGTGCGTAGAAGCGATGA |
| *ulaC* | CGCGTTACTACCAGGCGATTCT | GATGAGGATATCCACCGGATCG |
| *ulaD* | CGTCTTTCCGATATGGGCTTCA | CGATGGAACGTTTGAACTGACG |
| *ulaE* | CATGTGCCTTTCTGCTCATCGT | GCGTTTCGTTATTGGCTTCCTG |
| *ulaF* | TGCGACGCATCTCGAACTCTAC | TCAGTTCATACTCGCCCTGCAC |
| *uxaA* | GATAACGTCGCGGTCGCTTTAG | ATCCGCCAATGCATAACCAATC |
| *uxaB* | TGTTCTACGCTGGTGGACCGTA | CAGCACGTTGAGCGGATATTTG |
| *uxaC* | TCTGTGTACCGGTGATGCGTCT | AGTTATCCTGCGCCAGCAGTTC |
| *uxuA* | TCGTAGAAAGCGTGCCAATTCA | ATTCGAGGTCAGTACGGGTCCA |
| *uxuB* | TCCGTCAGTGGGTGATTGAAGA | TAAGCCGGGTTAGTCACGGTGT |
| *uxuR* | TTGCAACTGGAAGAGCGTGAAC | GTCATCCAGATGGCTGTGCAAC |
| *xseA* | GGCTGCTGCAACAGAAGTACGA | AGATGATCACCGGCAGAGAAGG |
| *xylA* | ACAATCCCGACGAACTGGTGTT | TGCGACATCTGCTTTACGCTTC |
| *xylB* | GCAGGCTTGCGACTTATCTCGT | TCAACCATTCCCACACCAACTG |
| *xylF* | GCCGGTAGATAACAACGCCAAG | GGCATCGTTTGAGGCAACTACA |
| *xylG* | ATATTACCCTCGCCGCACTCAA | TGCGCGGGTTAAGTAACAGACA |
| *xylH* | GGGTTTACAGTCTCCGGCCTCT | GCCATAAACATTCCGCCGAGTA |
| *xylR* | ACCCACCCGTTCATTACATTGC | AAATGCATATTCGCGCTCAGTG |
| *yaeJ* | ATTCCCGATGGTGAGCTTGAGA | CTTCGCGGTTCAGTTCCTGACT |
| *yaeQ* | TAAAGCGACGGTTAATGTGGCC | AACCACGGGTAAATTGCAGACG |
| *ybiS* | GCCTGCGTGTAAGTCATGGTTG | CGGTGGTAGACAGCGGGTTATG |
| *ybiT* | GGCGAAATGGACGGTTACTCTG | GGTGTCGATGTCGAGGTTGTTG |
| *ycfQ* | CACGCTATACGCGGAATTTACCA | TAAAGCAGTTGGCGATGGCAG |
| *ychH* | GGGTCTGGTGGTAATGGTGGTC | GTAACGGTCGCACACCTGTTCA |
| *yeiP* | TCCGTACCGGGCTGAAAGTAGA | GCCCTCTGGCATAAACAGCAAC |
| *yfiD* | TGCAGTAAGCAAACTGGGTGACA | TAACCGGATACACGGATGGTCAG |
| *yhcH* | CATTAGCTGCCAGACCGCAAG | CTGCCATGCCAAACAGAATCC |
| *yhfA* | CCACGCCTGTTTACGCACATTA | GCGGCAACCACTTCATACGAG |
| *yiaJ* | TAACTTCTCCAGCCGCGAAGAC | TGACTTCACGTAGTCCGGGTGA |
| *yiaK* | CGATGGTGGCTTTGATGATGAG | CGTATTCGTCGCTGTTGTCCTG |
| *yiaL* | GTAAACCGTCCGGAAGTGCATC | CGTAGCTGCCCGGTATCATTTC |
| *yiaM* | ATAACGCCCACGTTCAGGTCAC | CGGTGAATAATCGCTCCAGTCC |
| *yiaN* | GCGCTGTTTCTTCCAGTCATCA | CGAGGCCACCAGAAACATAACC |
| *yiaO* | GCGGGTAACTATCAGCGCAAAC | CTCCCTGCGGCACATCTTTAAC |
| *yjcH* | GCGCATTTCAGGGAGTTAGTCG | CAATTGGAATACCGCGTGTGAC |
| *ynfK* | CCGAAGGGCTGCGTAATAAAGA | GGCCGTTTGAAATGAGGGTGTA |
| *zraR* | CTGAATGTGGTGGCGATTGAAG | TATTTCCCGGCCAGTCGTAATG |
| *zraS* | TTAAACCGCGTGGTAAGCGAGT | GTTGGCGGTAAAGCGTAACTGG |
