# Supplementary material for: Improving Acetate Tolerance of Escherichia coli by Rewiring Its Global Regulator cAMP Receptor Protein (CRP)
Source: PLoS One. 2013 Oct 4;8(10):e77422. doi: 10.1371/journal.pone.0077422 (PMC3790751; doi:10.1371/journal.pone.0077422)
Supplement: Table S3 — CRP-regulated genes with >2-fold change in their expression level in A2 as compared to the control in the absence of sodium acetate stress, using a p-value threshold less than 0.05. (DOC) [file pone.0077422.s005.doc]

| **b-number** | **Gene** | **Function^a^** | **Fold-change^b^** | ***p*-value** |
| --- | --- | --- | --- | --- |
| **Up-regulated genes** | | | | |
| b1521 | *uxaB* | altronate oxidoreductase | 3.236 | 1.02E-03 |
| b2579 | *yfiD* | stress-induced alternate pyruvate formate-lyase subunit | 17.682 | 4.96E-04 |
| b0903 | *pflB* | pyruvate formate-lyase (inactive) | 15.511 | 3.01E-04 |
| b0904 | *focA* | FocA formate transporter | 6.872 | 7.16E-08 |
| b3172 | *argG* | argininosuccinate synthase | 5.528 | 6.10E-09 |
| b3870 | *glnA* | glutamine synthetase | 5.089 | 4.61E-05 |
| b1272 | *sohB* | predicted inner membrane peptidase | 5.068 | 2.94E-03 |
| b3868 | *glnG* | NtrC transcriptional dual regulator | 5.053 | 1.31E-05 |
| b3721 | *bglB* | 6-phospho-β-glucosidase B | 4.383 | 3.61E-06 |
| b3366 | *nirD* | nitrite reductase, small subunit | 4.338 | 1.16E-10 |
| b3906 | *rhaR* | RhaR transcriptional activator | 4.236 | 8.55E-06 |
| b1593 | *ynfK* | predicted dethiobiotin synthetase | 4.026 | 3.89E-06 |
| b3902 | *rhaD* | rhamnulose-1-phosphate aldolase | 4.022 | 1.08E-05 |
| b4197 | *ulaE* | L-xylulose 5-phosphate 3-epimerase | 3.991 | 1.53E-05 |
| b4033 | *malF* | maltose ABC transporter - membrane subunit | 3.898 | 1.81E-05 |
| b1779 | *gapA* | glyceraldehyde 3-phosphate dehydrogenase-A complex | 3.876 | 4.56E-08 |
| b3452 | *ugpA* | glycerol-3-phosphate/glycerol-2-phosphate ABC transporter - putative membrane subunit | 3.872 | 2.34E-07 |
| b3571 | *malS* | α-amylase | 3.784 | 2.98E-06 |
| b3413 | *gntX* | protein involved in utilization of DNA as a carbon source | 3.759 | 2.88E-06 |
| b3221 | *yhcH* | conserved protein | 3.728 | 6.27E-06 |
| b4122 | *fumB* | fumarase B | 3.662 | 7.64E-07 |
| b2241 | *glpA* | glycerol-3-phosphate dehydrogenase (anaerobic), large subunit | 3.598 | 4.14E-05 |
| b3076 | *ebgA* | evolved β-D-galactosidase, α subunit | 3.570 | 5.47E-06 |
| b3451 | *ugpE* | glycerol-3-phosphate/glycerol-2-phosphate ABC transporter - putative membrane subunit | 3.566 | 4.18E-07 |
| b2469 | *narQ* | NarQ sensory histidine kinase | 3.554 | 2.10E-03 |
| b0063 | *araB* | L-ribulokinase | 3.542 | 2.79E-06 |
| b3565 | *xylA* | xylose isomerase | 3.537 | 4.84E-08 |
| b3367 | *nirC* | NirC nitrite FNT transporter | 3.536 | 1.82E-04 |
| b4194 | *ulaB* | L-ascorbate-specific enzyme IIB component of PTS | 3.534 | 1.75E-06 |
| b3365 | *nirB* | dimer of large subunit of nitrite reductase | 3.532 | 5.19E-07 |
| b3575 | *yiaK* | 2,3-diketo-L-gulonate reductase | 3.524 | 4.32E-07 |
| b3512 | *gadE* | GadE DNA-binding transcriptional activator | 3.523 | 1.14E-05 |
| b2488 | *hyfH* | hydrogenase 4, component H | 3.475 | 2.12E-04 |
| b3904 | *rhaB* | L-rhamnulose kinase | 3.471 | 1.38E-04 |
| b2702 | *srlA* | glucitol/sorbitol-specific enzyme IIC component of PTS | 3.454 | 3.13E-03 |
| b0037 | *caiC* | carnitine-CoA ligase | 3.438 | 1.01E-02 |
| b1388 | *paaA* | ring 1,2-phenylacetyl-CoA epoxidase, monooxygenase subunit | 3.437 | 1.88E-05 |
| b4035 | *malK* | maltose ABC transporter - ATP binding subunit | 3.426 | 3.02E-05 |
| b4139 | *aspA* | aspartate ammonia-lyase | 3.425 | 1.78E-05 |
| b3395 | *hofM* | protein involved in utilization of DNA as a carbon source | 3.400 | 1.27E-05 |
| b2242 | *glpB* | glycerol-3-phosphate dehydrogenase (anaerobic), membrane anchor subunit | 3.398 | 4.35E-07 |
| b3225 | *nanA* | N-acetylneuraminate lyase | 3.394 | 1.66E-04 |
| b3564 | *xylB* | xylulokinase | 3.388 | 1.60E-04 |
| b4037 | *malM* | maltose regulon periplasmic protein | 3.363 | 1.61E-04 |
| b3241 | *aaeA* | AaeAB Hydroxylated, Aromatic Carboxylic Acid Efflux Transport System Protein A | 3.354 | 9.66E-06 |
| b4266 | *idnO* | 5-keto-D-gluconate 5-reductase | 3.349 | 1.44E-05 |
| b3093 | *exuT* | ExuT hexuronate MFS transporter | 3.348 | 5.44E-06 |
| b2802 | *fucI* | L-fucose isomerase | 3.308 | 1.10E-06 |
| b4004 | *zraR* | ZraR transcriptional activator | 3.307 | 7.21E-06 |
| b3708 | *tnaA* | L-cysteine desulfhydrase / tryptophanase | 3.306 | 1.98E-04 |
| b4288 | *fecD* | ferric dicitrate ABC transporter - membrane subunit | 3.305 | 3.19E-03 |
| b0106 | *hofC* | protein transport protein HofC | 3.298 | 3.00E-03 |
| b3569 | *xylR* | XylR transcriptional activator | 3.274 | 3.88E-05 |
| b1622 | *malY* | bifunctional β-cystathionase, PLP-dependent and regulator of maltose regulon | 3.272 | 1.39E-03 |
| b3925 | *glpX* | fructose 1,6-bisphosphatase II | 3.269 | 1.13E-05 |
| b2482 | *hyfB* | hydrogenase 4, component B | 3.264 | 2.91E-03 |
| b4032 | *malG* | maltose ABC transporter - membrane subunit | 3.263 | 2.78E-04 |
| b3574 | *yiaJ* | YiaJ DNA-binding transcriptional repressor | 3.259 | 7.38E-06 |
| b2535 | *csiE* | stationary phase inducible protein | 3.238 | 7.67E-08 |
| b4291 | *fecA* | outer membrane receptor; citrate-dependent iron transport, outer membrane receptor | 3.220 | 8.89E-04 |
| b4123 | *dcuB* | DcuB dicarboxylate Dcu transporter | 3.215 | 2.07E-04 |
| b3514 | *mdtF* | MdtEF-TolC multidrug efflux transport system - permease subunit | 3.170 | 2.92E-06 |
| b3405 | *ompR* | DNA-binding response regulator in two-component regulatory system with EnvZ | 3.169 | 6.02E-05 |
| b4324 | *uxuR* | UxuR DNA-binding transcriptional repressor | 3.159 | 1.13E-05 |
| b3588 | *aldB* | acetaldehyde dehydrogenase | 3.143 | 3.63E-05 |
| b3583 | *sgbE* | L-ribulose-5-phosphate 4-epimerase | 3.131 | 1.21E-06 |
| b3566 | *xylF* | xylose ABC transporter - periplasmic binding protein | 3.130 | 2.40E-06 |
| b3831 | *udp* | uridine phosphorylase | 3.107 | 2.32E-05 |
| b3666 | *uhpT* | UhpT-hexose phosphate MFS transporter | 3.101 | 3.52E-06 |
| b4068 | *yjcH* | conserved inner membrane protein | 3.098 | 1.30E-06 |
| b3115 | *tdcD* | propionate kinase | 3.090 | 1.12E-03 |
| b3430 | *glgC* | glucose-1-phosphate adenylyltransferase | 3.073 | 8.72E-10 |
| b2483 | *hyfC* | hydrogenase 4, component C | 3.059 | 2.60E-05 |
| b0330 | *prpR* | PrpR DNA-binding transcriptional dual regulator | 3.059 | 2.16E-02 |
| b2926 | *pgk* | phosphoglycerate kinase | 3.054 | 6.29E-07 |
| b3580 | *lyxK* | L-xylulose kinase | 3.046 | 2.75E-03 |
| b3240 | *aaeB* | AaeAB Hydroxylated, Aromatic Carboxylic Acid Efflux Transport System Protein B | 3.038 | 4.68E-07 |
| b3723 | *bglG* | BglG transcriptional antiterminator | 3.036 | 3.71E-04 |
| b2801 | *fucP* | FucP fucose MFS transporter | 3.033 | 9.96E-06 |
| b0107 | *hofB* | protein involved in plasmid replication | 3.033 | 5.97E-06 |
| b3428 | *glgP* | glycogen phosphorylase | 2.999 | 1.55E-04 |
| b0062 | *araA* | L-arabinose isomerase | 2.994 | 3.61E-04 |
| b4323 | *uxuB* | D-mannonate oxidoreductase | 2.994 | 3.22E-04 |
| b1901 | *araF* | arabinose ABC transporter - periplasmic binding protein | 2.990 | 1.92E-04 |
| b4264 | *idnR* | IdnR-5-ketogluconate DNA-binding transcriptional dual regulator | 2.979 | 2.32E-06 |
| b3135 | *agaA* | predicted truncated N-acetylgalactosamine-6-phosphate deacetylase | 2.973 | 5.66E-08 |
| b3224 | *nanT* | NanT sialic acid MFS transporter | 2.969 | 3.63E-06 |
| b2805 | *fucR* | FucR transcriptional activator | 2.965 | 9.43E-07 |
| b4003 | *zraS* | ZraS sensory histidine kinase | 2.960 | 3.96E-05 |
| b4119 | *melA* | α-galactosidase | 2.957 | 1.25E-04 |
| b2660 | *lhgO* | L-2-hydroxyglutarate oxidase | 2.955 | 3.83E-06 |
| b3577 | *yiaM* | predicted transporter | 2.949 | 9.72E-05 |
| b4382 | *deoA* | thymidine phosphorylase | 2.929 | 1.27E-03 |
| b3418 | *malT* | MalT transcriptional activator | 2.917 | 1.61E-06 |
| b0110 | *ampD* | N-acetyl-anhydromuramyl-L-alanine-amidase | 2.913 | 2.97E-05 |
| b3567 | *xylG* | xylose ABC transporter - ATP binding subunit | 2.876 | 2.94E-05 |
| b1182 | *hlyE* | hemolysin E | 2.872 | 5.76E-03 |
| b0039 | *caiA* | crotonobetainyl-CoA reductase | 2.852 | 4.66E-03 |
| b2614 | *grpE* | phage lambda replication; host DNA synthesis; heat shock protein; protein repair | 2.851 | 8.35E-06 |
| b4067 | *actP* | acetate / glycolate transporter | 2.845 | 2.39E-04 |
| b3599 | *mtlA* | mannitol PTS permease | 2.830 | 8.51E-06 |
| b3081 | *fadH* | 2,4-dienoyl-CoA reductase | 2.815 | 3.55E-05 |
| b4240 | *treB* | fused trehalose(maltose)-specific PTS enzyme: IIB component/IIC component | 2.803 | 1.71E-04 |
| b4138 | *dcuA* | DcuA dicarboxylate Dcu transporter | 2.801 | 8.58E-04 |
| b4287 | *fecE* | ferric dicitrate ABC transporter - ATP binding subunit | 2.788 | 7.01E-06 |
| b4196 | *ulaD* | 3-keto-L-gulonate 6-phosphate decarboxylase | 2.786 | 3.64E-06 |
| b2092 | *gatC* | galactitol-specific enzyme IIC component of PTS | 2.782 | 9.45E-05 |
| b1392 | *paaE* | ring 1,2-phenylacetyl-CoA epoxidase, reductase subunit | 2.779 | 4.86E-05 |
| b3423 | *glpR* | GlpR DNA-binding transcriptional repressor | 2.777 | 7.26E-06 |
| b4120 | *melB* | MelB GPH transporter | 2.767 | 2.74E-04 |
| b1492 | *gadC* | GadC GABA APC transporter | 2.765 | 1.28E-03 |
| b4198 | *ulaF* | L-ribulose 5-phosphate 4-epimerase | 2.752 | 8.77E-04 |
| b3091 | *uxaA* | D-altronate dehydratase | 2.752 | 1.62E-03 |
| b0190 | *yaeQ* | conserved protein | 2.749 | 4.84E-06 |
| b4265 | *idnT* | L-idonate / 5-ketogluconate / gluconate transporter | 2.741 | 3.23E-04 |
| b3077 | *ebgC* | evolved β-D-galactosidase, β subunit | 2.738 | 2.95E-07 |
| b2943 | *galP* | GalP - galactose MFS transporter | 2.734 | 9.84E-07 |
| b3404 | *envZ* | EnvZ sensory histidine kinase | 2.727 | 8.89E-04 |
| b0061 | *araD* | L-ribulose 5-phosphate 4-epimerase | 2.724 | 2.36E-08 |
| b2663 | *gabP* | GabP APC transporter | 2.723 | 6.31E-06 |
| b3709 | *tnaB* | TnaB tryptophan ArAAP transporter | 2.720 | 2.73E-07 |
| b3907 | *rhaT* | rhamnose RhaT transporter | 2.716 | 8.68E-05 |
| b1384 | *feaR* | FeaR DNA-binding transcriptional activator | 2.712 | 7.91E-05 |
| b2799 | *fucO* | L-1,2-propanediol oxidoreductase | 2.710 | 9.63E-05 |
| b1517 | *lsrF* | predicted class I aldolase | 2.708 | 6.78E-05 |
| b0343 | *lacY* | LacY lactose MFS transporter | 2.708 | 3.55E-07 |
| b4321 | *gntP* | GntP Gluconate Gnt transporter | 2.705 | 3.84E-05 |
| b3392 | *hofP* | protein involved in utilization of DNA as a carbon source | 2.695 | 4.83E-05 |
| b4233 | *mpl* | UDP-N-acetylmuramate:L-alanyl-γ-D-glutamyl-meso-diaminopimelate ligase | 2.693 | 3.94E-04 |
| b3513 | *mdtE* | MdtEF-TolC multidrug efflux transport system - membrane fusion protein | 2.691 | 8.96E-07 |
| b3368 | *cysG* | uroporphyrin III C-methyltransferase [multifunctional] | 2.691 | 4.31E-06 |
| b1389 | *paaB* | ring 1,2-phenylacetyl-CoA epoxidase subunit | 2.682 | 1.72E-02 |
| b2957 | *ansB* | asparaginase II | 2.682 | 6.43E-06 |
| b3214 | *gltF* | periplasmic protein | 2.676 | 2.63E-06 |
| b1391 | *paaD* | phenylacetate degradation protein | 2.676 | 1.72E-02 |
| b2468 | *aegA* | putative oxidoreductase, Fe-S subunit | 2.666 | 2.89E-03 |
| b3600 | *mtlD* | mannitol-1-phosphate 5-dehydrogenase | 2.663 | 9.36E-06 |
| b4213 | *cpdB* | 2',3'-cyclic nucleotide 2'-phosphodiesterase / 3'-nucleotidase | 2.662 | 2.86E-03 |
| b4193 | *ulaA* | L-ascorbate-specific enzyme IIC component of PTS | 2.657 | 1.75E-03 |
| b0344 | *lacZ* | β-galactosidase | 2.643 | 2.18E-05 |
| b2841 | *araE* | AraE arabinose MFS transporter | 2.642 | 2.29E-05 |
| b3722 | *bglF* | β-glucoside PTS permease | 2.636 | 7.96E-05 |
| b3601 | *mtlR* | MtlR DNA-binding transcriptional repressor | 2.631 | 4.56E-04 |
| b3133 | *agaV* | PTS system, cytoplasmic, N-acetylgalactosamine-specific IIB component 2 (EIIB-AGA) | 2.631 | 1.33E-03 |
| b3517 | *gadA* | glutamate decarboxylase A | 2.623 | 2.85E-06 |
| b2481 | *hyfA* | hydrogenase 4, component A | 2.621 | 2.00E-05 |
| b3528 | *dctA* | DctA dicarboxylate DAACS transporter | 2.612 | 1.98E-04 |
| b4381 | *deoC* | 2-deoxyribose-5-phosphate aldolase, NAD(P)-linked | 2.611 | 1.19E-04 |
| b4384 | *deoD* | purine-nucleoside phosphorylase | 2.605 | 1.39E-04 |
| b3869 | *glnL* | NtrB sensory histidine kinase | 2.603 | 2.67E-05 |
| b2708 | *gutQ* | D-arabinose 5-phosphate isomerase | 2.593 | 8.20E-05 |
| b1615 | *uidC* | membrane-associated protein | 2.573 | 3.93E-05 |
| b2009 | *sbmC* | DNA gyrase inhibitor | 2.562 | 2.98E-06 |
| b2803 | *fucK* | L-fuculokinase | 2.560 | 3.27E-07 |
| b2925 | *fbaA* | fructose bisphosphate aldolase class II | 2.556 | 3.33E-08 |
| b0342 | *lacA* | galactoside O-acetyltransferase | 2.543 | 3.30E-05 |
| b2800 | *fucA* | L-fuculose-phosphate aldolase | 2.542 | 3.14E-07 |
| b1390 | *paaC* | ring 1,2-phenylacetyl-CoA epoxidase, structural subunit | 2.529 | 3.93E-03 |
| b3092 | *uxaC* | D-glucuronate isomerase / D-galacturonate isomerase | 2.524 | 9.98E-05 |
| b4195 | *ulaC* | L-ascorbate-specific enzyme IIA component of PTS | 2.519 | 5.66E-05 |
| b1656 | *sodB* | superoxide dismutase (Fe) | 2.518 | 4.38E-07 |
| b2487 | *hyfG* | hydrogenase 4, large subunit | 2.516 | 3.96E-04 |
| b2243 | *glpC* | glycerol-3-phosphate dehydrogenase (anaerobic), small subunit | 2.516 | 5.63E-05 |
| b4118 | *melR* | MelR DNA-binding transcriptional dual regulator | 2.514 | 6.03E-05 |
| b3113 | *tdcF* | predicted L-PSP (mRNA) endoribonuclease | 2.508 | 1.17E-04 |
| b2707 | *srlR* | GutR DNA-binding transcriptional repressor | 2.506 | 2.54E-07 |
| b2715 | *ascF* | β-glucoside PTS permease | 2.489 | 6.92E-05 |
| b4036 | *lamB* | phage lambda receptor protein; maltose high-affinity receptor | 2.476 | 4.51E-04 |
| b3117 | *tdcB* | catabolic threonine dehydratase | 2.475 | 5.64E-06 |
| b2147 | *preA* | NADH-dependent dihydropyrimidine dehydrogenase subunit | 2.471 | 1.03E-03 |
| b2964 | *nupG* | NupG nucleoside MFS transporter | 2.464 | 2.50E-04 |
| b0349 | *mhpC* | 2-hydroxy-6-ketonona-2,4-dienedioate hydrolase | 2.464 | 2.54E-04 |
| b3114 | *tdcE* | 2-ketobutyrate formate-lyase/pyruvate formate-lyase 4, inactive | 2.461 | 4.16E-05 |
| b1805 | *fadD* | fatty acyl-CoA synthetase | 2.461 | 1.96E-05 |
| b1530 | *marR* | MarR DNA-binding transcriptional repressor | 2.456 | 4.23E-03 |
| b3393 | *hofO* | protein involved in utilization of DNA as a carbon source | 2.455 | 3.22E-05 |
| b3905 | *rhaS* | RhaS transcriptional activator | 2.447 | 1.33E-04 |
| b1395 | *paaH* | 3-hydroxyadipyl-CoA dehydrogenase (NAD+) | 2.445 | 5.77E-05 |
| b1514 | *lsrC* | AI-2 ABC transporter - membrane subunit | 2.442 | 3.69E-03 |
| b1516 | *lsrB* | AI-2 ABC transporter - periplasmic binding protein | 2.428 | 2.09E-03 |
| b0108 | *ppdD* | prepilin peptidase dependent protein | 2.428 | 3.16E-07 |
| b1738 | *chbB* | N,N'-diacetylchitobiose-specific enzyme IIB component of PTS | 2.425 | 4.21E-05 |
| b2485 | *hyfE* | hydrogenase 4, component E | 2.423 | 4.39E-03 |
| b2091 | *gatD* | galactitol-1-phosphate dehydrogenase | 2.418 | 1.95E-03 |
| b1900 | *araG* | arabinose ABC transporter - ATP binding subunit | 2.413 | 2.48E-02 |
| b1002 | *agp* | 3-phytase / glucose-1-phosphatase | 2.410 | 1.58E-04 |
| b3243 | *aaeR* | quorum sensing transcriptional regulator of LYSR-type | 2.410 | 8.18E-05 |
| b2491 | *hyfR* | HyfR DNA-binding transcriptional activator | 2.401 | 3.62E-07 |
| b3222 | *nanK* | N-acetylmannosamine kinase | 2.400 | 1.76E-05 |
| b1518 | *lsrG* | Autoinducer 2-degrading protein | 2.397 | 1.03E-05 |
| b3429 | *glgA* | glycogen synthase | 2.387 | 4.20E-05 |
| b0677 | *nagA* | N-acetylglucosamine-6-phosphate deacetylase | 2.384 | 2.28E-05 |
| b3903 | *rhaA* | L-rhamnose isomerase | 2.381 | 1.94E-04 |
| b0765 | *modC* | molybdate ABC transporter - ATP binding subunit | 2.380 | 4.89E-06 |
| b3578 | *yiaN* | L-dehydroascorbate transporter | 2.379 | 1.91E-04 |
| b4239 | *treC* | trehalose-6-phosphate hydrolase | 2.377 | 3.26E-05 |
| b0679 | *nagE* | N-acetylglucosamine PTS permease | 2.371 | 8.52E-03 |
| b0064 | *araC* | AraC DNA-binding transcriptional dual regulator | 2.370 | 2.03E-05 |
| b1421 | *trg* | methyl-accepting chemotaxis protein III, ribose and galactose sensor receptor | 2.364 | 8.70E-03 |
| b2492 | *focB* | FocB formate FNT transporter | 2.363 | 3.79E-03 |
| b4268 | *idnK* | D-gluconate kinase, thermosensitive | 2.360 | 8.58E-06 |
| b0111 | *ampE* | predicted inner membrane protein | 2.354 | 5.92E-04 |
| b1736 | *chbA* | N,N'-diacetylchitobiose-specific enzyme IIA component of PTS | 2.351 | 5.74E-05 |
| b1387 | *paaZ* | oxepin-CoA hydrolase/3-oxo-5,6-dehydrosuberyl-CoA semialdehyde dehydrogenase | 2.351 | 6.31E-03 |
| b4383 | *deoB* | phosphopentomutase | 2.348 | 2.12E-04 |
| b4124 | *dcuR* | DcuR transcriptional activator | 2.330 | 1.36E-05 |
| b1519 | *tam* | trans-aconitate methyltransferase | 2.329 | 2.75E-04 |
| b0043 | *fixC* | flavoprotein (electron transport), possibly involved in anaerobic carnitine metabolism | 2.320 | 4.79E-03 |
| b2705 | *srlD* | sorbitol-6-phosphate dehydrogenase | 2.317 | 1.34E-03 |
| b4267 | *idnD* | L-idonate 5-dehydrogenase | 2.316 | 5.59E-04 |
| b3424 | *glpG* | intramembrane serine protease GlpG | 2.303 | 1.35E-06 |
| b1617 | *uidA* | β-D-glucuronidase | 2.303 | 6.06E-05 |
| b2703 | *srlE* | glucitol/sorbitol-specific enzyme IIB component of PTS | 2.297 | 2.29E-06 |
| b0113 | *pdhR* | PdhR DNA-binding transcriptional dual regulator | 2.295 | 1.23E-06 |
| b2965 | *speC* | ornithine decarboxylase, biosynthetic | 2.293 | 1.90E-05 |
| b1493 | *gadB* | glutamate decarboxylase B | 2.293 | 5.20E-07 |
| b3934 | *cytR* | CytR DNA-binding transcriptional repressor | 2.285 | 3.14E-04 |
| b2486 | *hyfF* | hydrogenase 4, component F | 2.285 | 2.50E-05 |
| b3167 | *rbfA* | 30S ribosome binding factor | 2.282 | 4.81E-05 |
| b2146 | *preT* | NADH-dependent dihydropyrimidine dehydrogenase subunit | 2.281 | 9.08E-04 |
| b4476 | *gntU* | GntU gluconate Gnt transporter | 2.271 | 2.17E-07 |
| b0191 | *yaeJ* | peptidyl-tRNA hydrolase, ribosome rescue factor | 2.270 | 2.02E-04 |
| b1039 | *csgE* | curli production assembly/transport component | 2.269 | 4.22E-03 |
| b1205 | *ychH* | stress-induced protein | 2.265 | 1.95E-05 |
| b0035 | *caiE* | predicted acyl transferase | 2.261 | 1.20E-07 |
| b1737 | *chbC* | N,N'-diacetylchitobiose-specific enzyme IIC component of PTS | 2.256 | 5.96E-06 |
| b0763 | *modA* | molybdate ABC transporter - periplasmic binding protein | 2.254 | 1.60E-05 |
| b3394 | *hofN* | protein involved in utilization of DNA as a carbon source | 2.250 | 4.45E-04 |
| b1190 | *dadX* | alanine racemase 2, PLP-binding | 2.247 | 3.97E-05 |
| b1513 | *lsrA* | AI-2 ABC transporter - ATP binding subunit | 2.246 | 4.77E-04 |
| b1393 | *paaF* | predicted 2,3-dehydroadipyl-CoA hydratase | 2.244 | 1.72E-03 |
| b4311 | *nanC* | N-acetylneuraminic acid outer membrane channel | 2.240 | 1.20E-03 |
| b3582 | *sgbU* | predicted L-xylulose 5-phosphate 3-epimerase | 2.240 | 4.21E-04 |
| b3072 | *aer* | aerotaxis sensor receptor, flavoprotein | 2.234 | 4.88E-05 |
| b3415 | *gntT* | GntT Gluconate Gnt transporter | 2.232 | 1.89E-05 |
| b3568 | *xylH* | xylose ABC transporter - membrane subunit | 2.226 | 7.86E-06 |
| b3753 | *rbsR* | RbsR DNA-binding transcriptional repressor | 2.226 | 2.95E-05 |
| b4034 | *malE* | maltose ABC transporter - periplasmic binding protein | 2.221 | 1.29E-03 |
| b4322 | *uxuA* | D-mannonate dehydratase | 2.220 | 1.14E-02 |
| b1620 | *malI* | MalI DNA-binding transcriptional repressor | 2.220 | 4.60E-04 |
| b3516 | *gadX* | GadX DNA-binding transcriptional dual regulator | 2.215 | 4.24E-04 |
| b2365 | *dsdX* | DsdX Gnt tranporter | 2.214 | 1.02E-02 |
| b0396 | *araJ* | putative arabinose efflux transporter | 2.212 | 2.55E-04 |
| b0146, | *sfsA* | predicted DNA-binding transcriptional regulator of maltose metabolism | 2.212 | 2.01E-06 |
| b1761 | *gdhA* | glutamate dehydrogenase | 2.205 | 1.94E-05 |
| b0352 | *mhpE* | 4-hydroxy-2-ketovalerate aldolase | 2.202 | 3.50E-04 |
| b1038 | *csgF* | curli assembly component | 2.193 | 5.78E-05 |
| b2149 | *mglA* | galactose ABC transporter - ATP binding subunit | 2.189 | 2.95E-05 |
| b0040 | *caiT* | L-carnitine/γ-butyrobetaine antiport | 2.188 | 7.29E-06 |
| b3134 | *agaW* | PTS system N-acetylgalactosameine-specific IIC component 2 | 2.185 | 2.85E-04 |
| b1819 | *manZ* | mannose PTS permease - ManZ subunit | 2.181 | 3.27E-04 |
| b1891 | *flhC* | DNA-binding transcriptional dual regulator with FlhD | 2.179 | 7.44E-03 |
| b3118 | *tdcA* | TdcA DNA-binding transcriptional activator | 2.179 | 1.54E-03 |
| b1818 | *manY* | mannose PTS permease - ManY subunit | 2.175 | 2.53E-03 |
| b3453 | *ugpB* | glycerol-3-phosphate/glycerol-2-phosphate ABC transporter - putative periplasmic binding protein | 2.170 | 3.25E-05 |
| b1512 | *lsrR* | LsrR DNA-binding transcriptional repressor | 2.167 | 1.69E-04 |
| b1616 | *uidB* | UidB glucuronides GPH transporter | 2.164 | 1.96E-02 |
| b4460 | *araH* | arabinose ABC transporter - membrane subunit | 2.161 | 3.14E-03 |
| b2508 | *guaB* | IMP dehydrogenase | 2.155 | 4.44E-04 |
| b3450 | *ugpC* | glycerol-3-phosphate/glycerol-2-phosphate ABC transporter - putative ATP binding subunit | 2.153 | 3.47E-04 |
| b3356 | *yhfA* | conserved protein | 2.150 | 6.48E-07 |
| b3260 | *dusB* | tRNA dihydrouridine synthase | 2.140 | 9.67E-04 |
| b4471 | *tdcG* | L-serine deaminase III | 2.134 | 2.77E-05 |
| b3576 | *yiaL* | conserved protein | 2.119 | 9.52E-04 |
| b1396 | *paaI* | hydroxyphenylacetyl-CoA thioesterase | 2.119 | 9.91E-03 |
| b0348 | *mhpB* | 2,3-dihydroxyphenylpropionate 1,2-dioxygenase | 2.114 | 1.74E-05 |
| b1015 | *putP* | proline:sodium symporter | 2.108 | 1.24E-04 |
| b3579 | *yiaO* | L-dehydroascorbate transporter, periplasmic binding protein | 2.106 | 4.23E-05 |
| b2804 | *fucU* | L-fucose mutarotase | 2.104 | 3.52E-06 |
| b2716 | *ascB* | 6-phospho-β-glucosidase | 2.093 | 6.75E-05 |
| b2489 | *hyfI* | hydrogenase 4, small subunit | 2.088 | 1.78E-04 |
| b3116 | *tdcC* | TdcC threonine STP transporter | 2.084 | 6.94E-05 |
| b0678 | *nagB* | glucosamine-6-phosphate deaminase | 2.077 | 1.83E-04 |
| b1734 | *chbF* | diacetylchitobiose-6-phosphate hydrolase | 2.073 | 6.20E-05 |
| b4310 | *nanM* | N-acetylneuraminate mutarotase | 2.073 | 2.54E-04 |
| b2231 | *gyrA* | DNA gyrase, subunit A | 2.071 | 3.46E-04 |
| b2659 | *csiD* | predicted protein | 2.070 | 2.04E-04 |
| b2148 | *mglC* | galactose ABC transporter - membrane subunit | 2.057 | 1.86E-03 |
| b2706 | *gutM* | GutM DNA-binding transcriptional activator | 2.057 | 9.45E-06 |
| b2661 | *gabD* | succinate semialdehyde dehydrogenase, NADP+-dependent | 2.048 | 6.65E-03 |
| b1511 | *lsrK* | autoinducer-2 kinase | 2.045 | 3.64E-06 |
| b1394 | *paaG* | predicted ring 1,2-epoxyphenylacetyl-CoA isomerase (oxepin-CoA forming) | 2.043 | 4.99E-03 |
| b1735 | *chbR* | ChbR DNA-binding transcriptional dual regulator | 2.040 | 1.52E-02 |
| b3749 | *rbsA* | ribose ABC transporter - putative ATP binding subunit | 2.038 | 2.86E-03 |
| b3132 | *kbaZ* | tagatose 6-phosphate aldolase 1, kbaZ subunit | 2.031 | 2.31E-06 |
| b1892 | *flhD* | DNA-binding transcriptional dual regulator with FlhC | 2.024 | 1.02E-05 |
| b4290 | *fecB* | ferric dicitrate ABC transporter - periplasmic binding protein | 2.010 | 1.60E-03 |
| b2171 | *yeiP* | predicted dehydrogenase, NAD-dependent | 2.005 | 6.33E-04 |
| b3581 | *sgbH* | 3-keto-L-gulonate 6-phosphate decarboxylase | 2.004 | 1.27E-03 |
| b4016 | *aceK* | isocitrate dehydrogenase phosphatase / isocitrate dehydrogenase kinase | 2.000 | 7.03E-05 |
| **Down-regulated genes** | | | | |
| b0759 | *galE* | UDP-glucose 4-epimerase | -143.896 | 4.30E-02 |
| b0726 | *sucA* | 2-oxoglutarate decarboxylase, thiamin-requiring | -5.100 | 1.74E-08 |
| b0720 | *gltA* | citrate synthase | -4.920 | 4.21E-10 |
| b0728 | *sucC* | succinyl-CoA synthetase, β subunit | -4.302 | 2.04E-06 |
| b0929 | *ompF* | outer membrane porin F | -3.973 | 5.50E-07 |
| b0723 | *sdhA* | succinate dehydrogenase flavoprotein | -3.841 | 3.33E-05 |
| b0721 | *sdhC* | succinate dehydrogenase membrane protein | -3.831 | 9.56E-08 |
| b0722 | *sdhD* | succinate dehydrogenase membrane protein | -3.783 | 1.02E-04 |
| b0727 | *sucB* | dihydrolipoyltranssuccinylase | -3.428 | 1.23E-07 |
| b0118 | *acnB* | bifunctional aconitate hydratase 2 and 2-methylisocitrate dehydratase | -3.189 | 4.55E-08 |
| b0729 | *sucD* | succinyl-CoA synthetase, α subunit | -2.718 | 2.32E-05 |
| b4014 | *aceB* | malate synthase A | -2.653 | 6.60E-03 |
| b3236 | *mdh* | malate dehydrogenase | -2.185 | 5.09E-04 |
| b4015 | *aceA* | isocitrate lyase | -2.037 | 1.22E-02 |

^a^From the EcoCyc database (http://ecocyc.org)

^b^Fold-change in the gene expression level between A2 and the control (average of duplicate experiments). Statistics were calculated from duplicate samples.
