# Supplementary material for: Improving Acetate Tolerance of Escherichia coli by Rewiring Its Global Regulator cAMP Receptor Protein (CRP)
Source: PLoS One. 2013 Oct 4;8(10):e77422. doi: 10.1371/journal.pone.0077422 (PMC3790751; doi:10.1371/journal.pone.0077422)
Supplement: Table S4 — CRP-regulated genes with >2-fold change in their expression level in A2 as compared to the control in the presence of sodium acetate stress, using a p-value threshold less than 0.05. (DOC) [file pone.0077422.s006.doc]

| **b-number** | **Gene** | **Function^a^** | **Fold-change^b^** | ***p*-value** |
| --- | --- | --- | --- | --- |
| **Up-regulated genes** | | | | |
| b0759 | *galE* | UDP-glucose 4-epimerase | 14.296 | 3.04E-03 |
| b0903 | *pflB* | pyruvate formate-lyase (inactive) | 4.405 | 5.38E-09 |
| b3517 | *gadA* | glutamate decarboxylase A | 3.128 | 1.72E-05 |
| b2925 | *fbaA* | fructose bisphosphate aldolase class II | 2.117 | 1.30E-06 |
| b2579 | *yfiD* | stress-induced alternate pyruvate formate-lyase subunit | 2.067 | 5.65E-05 |
| b3870 | *glnA* | glutamine synthetase | 2.016 | 1.49E-04 |
| **Down-regulated genes** | | | | |
| b1521 | *uxaB* | altronate oxidoreductase | -22.046 | 1.38E-04 |
| b1900 | *araG* | arabinose ABC transporter - ATP binding subunit | -27.339 | 1.16E-02 |
| b1516 | *lsrB* | AI-2 ABC transporter - periplasmic binding protein | -23.781 | 9.83E-07 |
| b1515 | *lsrD* | AI-2 ABC transporter - membrane subunit | -22.929 | 3.89E-06 |
| b1384 | *feaR* | FeaR DNA-binding transcriptional activator | -22.765 | 6.16E-03 |
| b1389 | *paaB* | ring 1,2-phenylacetyl-CoA epoxidase subunit | -21.973 | 4.46E-05 |
| b0350 | *mhpD* | 2-oxopent-4-enoate hydratase | -21.436 | 8.67E-08 |
| b1395 | *paaH* | 3-hydroxyadipyl-CoA dehydrogenase (NAD+) | -21.272 | 1.04E-06 |
| b1387 | *paaZ* | oxepin-CoA hydrolase/3-oxo-5,6-dehydrosuberyl-CoA semialdehyde dehydrogenase | -20.801 | 1.14E-03 |
| b1513 | *lsrA* | AI-2 ABC transporter - ATP binding subunit | -20.585 | 2.91E-03 |
| b1734 | *chbF* | diacetylchitobiose-6-phosphate hydrolase | -19.524 | 8.71E-06 |
| b1617 | *uidA* | β-D-glucuronidase | -19.391 | 9.65E-04 |
| b0348 | *mhpB* | 2,3-dihydroxyphenylpropionate 1,2-dioxygenase | -19.256 | 1.88E-04 |
| b4460 | *araH* | arabinose ABC transporter - membrane subunit | -19.062 | 1.18E-03 |
| b2486 | *hyfF* | hydrogenase 4, component F | -18.926 | 2.96E-04 |
| b1518 | *lsrG* | Autoinducer 2-degrading protein | -18.782 | 4.11E-07 |
| b1615 | *uidC* | membrane-associated protein | -18.666 | 2.33E-04 |
| b1390 | *paaC* | ring 1,2-phenylacetyl-CoA epoxidase, structural subunit | -17.576 | 3.51E-04 |
| b2492 | *focB* | FocB formate FNT transporter | -17.410 | 5.93E-04 |
| b2429 | *murP* | N-acetylmuramic acid permease, EIIBC component, PTS system | -17.258 | 9.94E-04 |
| b1620 | *malI* | MalI DNA-binding transcriptional repressor | -17.083 | 6.79E-04 |
| b0345 | *lacI* | LacI DNA-binding transcriptional repressor | -16.970 | 4.74E-04 |
| b1415 | *aldA* | aldehyde dehydrogenase A, NAD-linked | -16.883 | 1.13E-03 |
| b2490 | *hyfJ* | hydrogenase 4 component J, putative protein processing element | -16.816 | 8.67E-04 |
| b2469 | *narQ* | NarQ sensory histidine kinase | -16.771 | 6.14E-04 |
| b1737 | *chbC* | N,N'-diacetylchitobiose-specific enzyme IIC component of PTS | -16.763 | 2.33E-06 |
| b0343 | *lacY* | LacY lactose MFS transporter | -16.747 | 8.37E-06 |
| b2489 | *hyfI* | hydrogenase 4, small subunit | -16.681 | 8.96E-04 |
| b2704 | *srlB* | glucitol/sorbitol-specific enzyme IIA component of PTS | -16.664 | 7.69E-08 |
| b1736 | *chbA* | N,N'-diacetylchitobiose-specific enzyme IIA component of PTS | -16.238 | 1.18E-04 |
| b1388 | *paaA* | ring 1,2-phenylacetyl-CoA epoxidase, monooxygenase subunit | -16.178 | 3.51E-06 |
| b1616 | *uidB* | UidB glucuronides GPH transporter | -16.029 | 6.33E-09 |
| b1038 | *csgF* | curli assembly component | -15.996 | 1.27E-03 |
| b2146 | *preT* | NADH-dependent dihydropyrimidine dehydrogenase subunit | -15.948 | 1.39E-05 |
| b1112 | *bhsA* | protein involved in stress resistance and biofilm formation | -15.900 | 1.42E-04 |
| b1039 | *csgE* | curli production assembly/transport component | -15.872 | 1.20E-04 |
| b1517 | *lsrF* | predicted class I aldolase | -15.718 | 5.63E-04 |
| b0349 | *mhpC* | 2-hydroxy-6-ketonona-2,4-dienedioate hydrolase | -15.381 | 5.77E-05 |
| b0037 | *caiC* | carnitine-CoA ligase | -15.068 | 3.11E-02 |
| b1621 | *malX* | fused maltose and glucose-specific PTS enzymes: IIB component, IIC component | -14.998 | 3.35E-05 |
| b1735 | *chbR* | ChbR DNA-binding transcriptional dual regulator | -14.995 | 3.91E-07 |
| b1901 | *araF* | arabinose ABC transporter - periplasmic binding protein | -14.914 | 2.82E-03 |
| b1398 | *paaK* | phenylacetate-CoA ligase | -14.818 | 2.67E-07 |
| b2487 | *hyfG* | hydrogenase 4, large subunit | -14.592 | 3.01E-03 |
| b1037 | *csgG* | curli production component | -14.578 | 8.49E-04 |
| b2482 | *hyfB* | hydrogenase 4, component B | -14.508 | 1.96E-06 |
| b2366 | *dsdA* | D-serine deaminase | -14.464 | 2.08E-03 |
| b1805 | *fadD* | fatty acyl-CoA synthetase | -14.394 | 3.83E-06 |
| b2150 | *mglB* | galactose ABC transporter - periplasmic binding protein | -14.359 | 1.65E-04 |
| b0765 | *modC* | molybdate ABC transporter - ATP binding subunit | -14.267 | 1.77E-03 |
| b1512 | *lsrR* | LsrR DNA-binding transcriptional repressor | -14.183 | 2.47E-03 |
| b2485 | *hyfE* | hydrogenase 4, component E | -14.106 | 1.41E-05 |
| b1738 | *chbB* | N,N'-diacetylchitobiose-specific enzyme IIB component of PTS | -14.105 | 6.30E-04 |
| b0107 | *hofB* | protein involved in plasmid replication | -14.098 | 4.90E-05 |
| b1531 | *marA* | MarA DNA-binding transcriptional dual regulator | -14.022 | 1.84E-08 |
| b1511 | *lsrK* | autoinducer-2 kinase | -13.914 | 1.28E-03 |
| b1530 | *marR* | MarR DNA-binding transcriptional repressor | -13.890 | 2.47E-04 |
| b2702 | *srlA* | glucitol/sorbitol-specific enzyme IIC component of PTS | -13.833 | 5.45E-05 |
| b1182 | *hlyE* | hemolysin E | -13.822 | 4.42E-04 |
| b1514 | *lsrC* | AI-2 ABC transporter - membrane subunit | -13.766 | 3.18E-09 |
| b2149 | *mglA* | galactose ABC transporter - ATP binding subunit | -13.734 | 7.15E-07 |
| b2151 | *galS* | GalS DNA-binding transcriptional dual regulator | -13.693 | 1.02E-03 |
| b1190 | *dadX* | alanine racemase 2, PLP-binding | -13.625 | 6.66E-06 |
| b2428 | *murQ* | N-acetylmuramic acid 6-phosphate etherase | -13.620 | 3.73E-04 |
| b0723 | *sdhA* | succinate dehydrogenase flavoprotein | -13.553 | 3.89E-03 |
| b0190 | *yaeQ* | conserved protein | -13.521 | 5.98E-04 |
| b0347 | *mhpA* | 3-(3-hydroxyphenyl)propanoate hydroxylase | -13.510 | 2.00E-06 |
| b1189 | *dadA* | D-amino acid dehydrogenase | -13.449 | 1.52E-07 |
| b1396 | *paaI* | hydroxyphenylacetyl-CoA thioesterase | -13.437 | 3.49E-04 |
| b0342 | *lacA* | galactoside O-acetyltransferase | -13.424 | 1.22E-08 |
| b0330 | *prpR* | PrpR DNA-binding transcriptional dual regulator | -13.411 | 7.29E-03 |
| b2491 | *hyfR* | HyfR DNA-binding transcriptional activator | -13.373 | 1.29E-03 |
| b0724 | *sdhB* | succinate dehydrogenase iron-sulfur protein | -13.366 | 2.32E-06 |
| b0722 | *sdhD* | succinate dehydrogenase membrane protein | -13.365 | 3.14E-04 |
| b2239 | *glpQ* | glycerophosphoryl diester phosphodiesterase, periplasmic | -13.352 | 1.37E-03 |
| b2242 | *glpB* | glycerol-3-phosphate dehydrogenase (anaerobic), membrane anchor subunit | -13.344 | 9.98E-05 |
| b2483 | *hyfC* | hydrogenase 4, component C | -13.331 | 6.76E-04 |
| b2092 | *gatC* | galactitol-specific enzyme IIC component of PTS | -13.308 | 2.62E-04 |
| b0344 | *lacZ* | β-galactosidase | -13.118 | 1.27E-04 |
| b0764 | *modB* | molybdate ABC transporter - membrane subunit | -13.098 | 2.51E-03 |
| b1393 | *paaF* | predicted 2,3-dehydroadipyl-CoA hydratase | -13.062 | 2.71E-06 |
| b2147 | *preA* | NADH-dependent dihydropyrimidine dehydrogenase subunit | -12.903 | 5.85E-05 |
| b2240 | *glpT* | GlpT glycerol-3-P MFS transporter | -12.784 | 2.07E-10 |
| b1392 | *paaE* | ring 1,2-phenylacetyl-CoA epoxidase, reductase subunit | -12.778 | 6.56E-04 |
| b2243 | *glpC* | glycerol-3-phosphate dehydrogenase (anaerobic), small subunit | -12.741 | 3.76E-03 |
| b3115 | *tdcD* | propionate kinase | -12.737 | 6.99E-04 |
| b1594 | *dgsA* | DgsA DNA-binding transcriptional repressor | -12.727 | 1.95E-04 |
| b1391 | *paaD* | phenylacetate degradation protein | -12.722 | 1.07E-04 |
| b0035 | *caiE* | predicted acyl transferase | -12.714 | 1.68E-04 |
| b1891 | *flhC* | DNA-binding transcriptional dual regulator with FlhD | -12.639 | 4.64E-03 |
| b0063 | *araB* | L-ribulokinase | -12.614 | 9.26E-07 |
| b0111 | *ampE* | predicted inner membrane protein | -12.597 | 3.87E-07 |
| b0189 | *rof* | modulator of Rho-dependent transcription termination | -12.596 | 1.94E-11 |
| b0352 | *mhpE* | 4-hydroxy-2-ketovalerate aldolase | -12.518 | 7.83E-08 |
| b0721 | *sdhC* | succinate dehydrogenase membrane protein | -12.484 | 9.28E-10 |
| b2484 | *hyfD* | hydrogenase 4, component D | -12.400 | 8.67E-04 |
| b4034 | *malE* | maltose ABC transporter - periplasmic binding protein | -12.188 | 3.73E-03 |
| b4471 | *tdcG* | L-serine deaminase III | -12.157 | 1.09E-03 |
| b1733 | *chbG* | conserved protein | -12.116 | 7.84E-04 |
| b0346 | *mhpR* | MhpR transcriptional activator | -12.086 | 5.07E-04 |
| b1002 | *agp* | 3-phytase / glucose-1-phosphatase | -12.074 | 2.42E-04 |
| b2344 | *fadL* | long-chain fatty acid outer membrane transporter; sensitivity to phage T2 | -12.040 | 1.28E-03 |
| b0763 | *modA* | molybdate ABC transporter - periplasmic binding protein | -12.038 | 2.31E-04 |
| b0396 | *araJ* | putative arabinose efflux transporter | -11.968 | 1.36E-06 |
| b1421 | *trg* | methyl-accepting chemotaxis protein III, ribose and galactose sensor receptor | -11.927 | 1.18E-05 |
| b2235 | *nrdB* | ribonucleoside diphosphate reductase 1, β subunit dimer | -11.881 | 3.77E-05 |
| b1612 | *fumA* | fumarase A | -11.873 | 7.22E-04 |
| b3114 | *tdcE* | 2-ketobutyrate formate-lyase/pyruvate formate-lyase 4, inactive | -11.806 | 2.05E-02 |
| b2148 | *mglC* | galactose ABC transporter - membrane subunit | -11.775 | 8.57E-04 |
| b2488 | *hyfH* | hydrogenase 4, component H | -11.649 | 1.57E-05 |
| b0108 | *ppdD* | prepilin peptidase dependent protein | -11.559 | 2.19E-04 |
| b0064 | *araC* | AraC DNA-binding transcriptional dual regulator | -11.548 | 1.13E-10 |
| b1622 | *malY* | bifunctional β-cystathionase, PLP-dependent and regulator of maltose regulon | -11.544 | 5.84E-04 |
| b0106 | *hofC* | protein transport protein HofC | -11.496 | 7.43E-08 |
| b1892 | *flhD* | DNA-binding transcriptional dual regulator with FlhC | -11.281 | 8.74E-04 |
| b0036 | *caiD* | crotonobetainyl-CoA hydratase | -11.280 | 1.66E-06 |
| b0062 | *araA* | L-arabinose isomerase | -11.225 | 8.82E-06 |
| b1394 | *paaG* | predicted ring 1,2-epoxyphenylacetyl-CoA isomerase (oxepin-CoA forming) | -11.187 | 6.28E-05 |
| b1015 | *putP* | proline:sodium symporter | -11.180 | 1.14E-04 |
| b2091 | *gatD* | galactitol-1-phosphate dehydrogenase | -11.066 | 5.05E-04 |
| b2468 | *aegA* | putative oxidoreductase, Fe-S subunit | -11.000 | 2.48E-04 |
| b3575 | *yiaK* | 2,3-diketo-L-gulonate reductase | -10.916 | 5.81E-03 |
| b1519 | *tam* | trans-aconitate methyltransferase | -10.776 | 1.59E-04 |
| b2804 | *fucU* | L-fucose mutarotase | -10.709 | 2.37E-06 |
| b1593 | *ynfK* | predicted dethiobiotin synthetase | -10.694 | 6.39E-04 |
| b0805 | *fiu* | putative outer membrane receptor for iron transport | -10.648 | 7.15E-07 |
| b1040 | *csgD* | CsgD DNA-binding transcriptional dual regulator | -10.623 | 6.96E-07 |
| b0040 | *caiT* | L-carnitine/γ-butyrobetaine antiport | -10.583 | 4.21E-04 |
| b1111 | *ycfQ* | predicted DNA-binding transcriptional regulator | -10.559 | 4.18E-04 |
| b0929 | *ompF* | outer membrane porin F | -10.495 | 1.23E-03 |
| b0039 | *caiA* | crotonobetainyl-CoA reductase | -10.329 | 1.18E-06 |
| b0679 | *nagE* | N-acetylglucosamine PTS permease | -10.274 | 5.15E-07 |
| b2802 | *fucI* | L-fucose isomerase | -10.253 | 3.29E-04 |
| b3528 | *dctA* | DctA dicarboxylate DAACS transporter | -10.145 | 4.88E-03 |
| b2716 | *ascB* | 6-phospho-β-glucosidase | -10.098 | 4.44E-03 |
| b0720 | *gltA* | citrate synthase | -10.064 | 6.65E-07 |
| b2481 | *hyfA* | hydrogenase 4, component A | -10.033 | 4.33E-06 |
| b0042 | *fixB* | probable flavoprotein subunit required for anaerobic carnitine metabolism | -9.999 | 1.77E-05 |
| b3723 | *bglG* | BglG transcriptional antiterminator | -9.980 | 1.19E-04 |
| b0034 | *caiF* | CaiF transcriptional activator | -9.944 | 2.68E-04 |
| b4193 | *ulaA* | L-ascorbate-specific enzyme IIC component of PTS | -9.884 | 7.13E-03 |
| b2801 | *fucP* | FucP fucose MFS transporter | -9.837 | 1.45E-04 |
| b4120 | *melB* | MelB GPH transporter | -9.825 | 5.93E-03 |
| b4197 | *ulaE* | L-xylulose 5-phosphate 3-epimerase | -9.809 | 8.56E-03 |
| b4288 | *fecD* | ferric dicitrate ABC transporter - membrane subunit | -9.774 | 9.20E-03 |
| b2365 | *dsdX* | DsdX Gnt tranporter | -9.762 | 8.39E-05 |
| b3580 | *lyxK* | L-xylulose kinase | -9.726 | 1.07E-03 |
| b3709 | *tnaB* | TnaB tryptophan ArAAP transporter | -9.658 | 2.47E-03 |
| b2155 | *cirA* | outer membrane receptor involved in uptake of ferric dihyroxybenzoylserine | -9.599 | 5.32E-04 |
| b2232 | *ubiG* | bifunctional 3-demethylubiquinone-8 3-O-methyltransferase and 2-octaprenyl-6-hydroxyphenol methylase | -9.594 | 1.15E-05 |
| b0041 | *fixA* | probable flavoprotein subunit required for anaerobic carnitine metabolism | -9.543 | 2.96E-04 |
| b0110 | *ampD* | N-acetyl-anhydromuramyl-L-alanine-amidase | -9.479 | 4.72E-07 |
| b2663 | *gabP* | GabP APC transporter | -9.471 | 7.27E-04 |
| b0038 | *caiB* | γ-butyrobetainyl-CoA:carnitine CoA transferase | -9.424 | 3.39E-04 |
| b3221 | *yhcH* | conserved protein | -9.419 | 2.10E-03 |
| b0061 | *araD* | L-ribulose 5-phosphate 4-epimerase | -9.394 | 4.14E-07 |
| b0758 | *galT* | galactose-1-phosphate uridylyltransferase | -9.342 | 1.33E-04 |
| b3904 | *rhaB* | L-rhamnulose kinase | -9.321 | 5.19E-03 |
| b4322 | *uxuA* | D-mannonate dehydratase | -9.320 | 1.37E-04 |
| b4032 | *malG* | maltose ABC transporter - membrane subunit | -9.304 | 2.92E-03 |
| b4213 | *cpdB* | 2',3'-cyclic nucleotide 2'-phosphodiesterase / 3'-nucleotidase | -9.298 | 1.43E-04 |
| b4033 | *malF* | maltose ABC transporter - membrane subunit | -9.298 | 4.21E-04 |
| b4194 | *ulaB* | L-ascorbate-specific enzyme IIB component of PTS | -9.251 | 1.45E-02 |
| b4123 | *dcuB* | DcuB dicarboxylate Dcu transporter | -9.247 | 5.63E-03 |
| b3571 | *malS* | α-amylase | -9.245 | 4.07E-03 |
| b4198 | *ulaF* | L-ribulose 5-phosphate 4-epimerase | -9.232 | 1.04E-02 |
| b1272 | *sohB* | predicted inner membrane peptidase | -9.231 | 3.99E-05 |
| b2393 | *nupC* | NupC nucleoside NUP transporter | -9.163 | 3.13E-03 |
| b2234 | *nrdA* | ribonucleoside diphosphate reductase 1, α subunit dimer | -9.136 | 5.96E-04 |
| b3934 | *cytR* | CytR DNA-binding transcriptional repressor | -9.114 | 3.47E-03 |
| b3116 | *tdcC* | TdcC threonine STP transporter | -9.026 | 5.67E-03 |
| b0043 | *fixC* | flavoprotein (electron transport), possibly involved in anaerobic carnitine metabolism | -9.019 | 4.46E-08 |
| b3925 | *glpX* | fructose 1,6-bisphosphatase II | -9.003 | 2.73E-04 |
| b3753 | *rbsR* | RbsR DNA-binding transcriptional repressor | -9.002 | 6.81E-05 |
| b3566 | *xylF* | xylose ABC transporter - periplasmic binding protein | -8.981 | 3.36E-03 |
| b3453 | *ugpB* | glycerol-3-phosphate / glycerol-2-phosphate ABC transporter - putative periplasmic binding protein | -8.973 | 2.20E-03 |
| b3134 | *agaW* | PTS system N-acetylgalactosameine-specific IIC component 2 | -8.967 | 6.81E-03 |
| b2241 | *glpA* | glycerol-3-phosphate dehydrogenase (anaerobic), large subunit | -8.954 | 1.14E-03 |
| b3452 | *ugpA* | glycerol-3-phosphate / glycerol-2-phosphate ABC transporter - putative membrane subunit | -8.936 | 3.26E-03 |
| b3081 | *fadH* | 2,4-dienoyl-CoA reductase | -8.911 | 1.49E-02 |
| b2706 | *gutM* | GutM DNA-binding transcriptional activator | -8.884 | 7.84E-07 |
| b2805 | *fucR* | FucR transcriptional activator | -8.883 | 6.31E-04 |
| b3579 | *yiaO* | L-dehydroascorbate transporter, periplasmic binding protein | -8.881 | 3.55E-03 |
| b4196 | *ulaD* | 3-keto-L-gulonate 6-phosphate decarboxylase | -8.868 | 4.37E-03 |
| b2803 | *fucK* | L-fuculokinase | -8.859 | 1.04E-03 |
| b0146, | *sfsA* | predicted DNA-binding transcriptional regulator of maltose metabolism | -8.856 | 8.68E-10 |
| b3113 | *tdcF* | predicted L-PSP (mRNA) endoribonuclease | -8.798 | 2.80E-03 |
| b2800 | *fucA* | L-fuculose-phosphate aldolase | -8.784 | 8.79E-04 |
| b2660 | *lhgO* | L-2-hydroxyglutarate oxidase | -8.749 | 9.25E-04 |
| b0757 | *galK* | galactokinase | -8.738 | 1.25E-06 |
| b3091 | *uxaA* | D-altronate dehydratase | -8.714 | 8.31E-05 |
| b4016 | *aceK* | isocitrate dehydrogenase phosphatase / isocitrate dehydrogenase kinase | -8.703 | 7.39E-03 |
| b3721 | *bglB* | 6-phospho-β-glucosidase B | -8.688 | 2.72E-03 |
| b3568 | *xylH* | xylose ABC transporter - membrane subunit | -8.602 | 4.68E-03 |
| b2535 | *csiE* | stationary phase inducible protein | -8.547 | 1.31E-03 |
| b2715 | *ascF* | β-glucoside PTS permease | -8.529 | 3.88E-03 |
| b3569 | *xylR* | XylR transcriptional activator | -8.527 | 5.07E-03 |
| b4291 | *fecA* | outer membrane receptor; citrate-dependent iron transport, outer membrane receptor | -8.522 | 1.10E-04 |
| b0595 | *entB* | apo-EntB multimer | -8.521 | 4.62E-04 |
| b3926 | *glpK* | glycerol kinase | -8.515 | 6.41E-03 |
| b1819 | *manZ* | mannose PTS permease - ManZ subunit | -8.509 | 2.02E-03 |
| b0124 | *gcd* | glucose dehydrogenase | -8.505 | 2.15E-03 |
| b0113 | *pdhR* | PdhR DNA-binding transcriptional dual regulator | -8.482 | 5.58E-05 |
| b4036 | *lamB* | phage lambda receptor protein; maltose high-affinity receptor | -8.433 | 8.73E-03 |
| b2009 | *sbmC* | DNA gyrase inhibitor | -8.354 | 3.48E-03 |
| b4067 | *actP* | acetate / glycolate transporter | -8.349 | 2.39E-03 |
| b3367 | *nirC* | NirC nitrite FNT transporter | -8.321 | 3.33E-03 |
| b2957 | *ansB* | asparaginase II | -8.295 | 2.24E-03 |
| b3907 | *rhaT* | rhamnose RhaT transporter | -8.262 | 3.01E-03 |
| b3903 | *rhaA* | L-rhamnose isomerase | -8.241 | 1.34E-03 |
| b4321 | *gntP* | GntP Gluconate Gnt transporter | -8.220 | 1.79E-04 |
| b2705 | *srlD* | sorbitol-6-phosphate dehydrogenase | -8.202 | 4.46E-04 |
| b4037 | *malM* | maltose regulon periplasmic protein | -8.160 | 4.43E-03 |
| b3906 | *rhaR* | RhaR transcriptional activator | -8.157 | 6.28E-03 |
| b0675 | *nagD* | ribonucleotide monophosphatase | -8.144 | 4.68E-09 |
| b3708 | *tnaA* | L-cysteine desulfhydrase / tryptophanase | -8.135 | 4.02E-03 |
| b2799 | *fucO* | L-1,2-propanediol oxidoreductase | -8.126 | 8.73E-04 |
| b3077 | *ebgC* | evolved β-D-galactosidase, β subunit | -8.114 | 3.56E-03 |
| b3588 | *aldB* | acetaldehyde dehydrogenase | -8.085 | 2.99E-04 |
| b1276 | *acnA* | aconitate hydratase 1 | -8.044 | 1.13E-03 |
| b3224 | *nanT* | NanT sialic acid MFS transporter | -8.030 | 9.47E-03 |
| b3225 | *nanA* | N-acetylneuraminate lyase | -8.015 | 4.09E-03 |
| b2707 | *srlR* | GutR DNA-binding transcriptional repressor | -7.934 | 3.67E-03 |
| b0430 | *cyoC* | cytochrome bo terminal oxidase subunit III | -7.931 | 4.19E-04 |
| b3132 | *kbaZ* | tagatose 6-phosphate aldolase 1, kbaZ subunit | -7.928 | 3.04E-03 |
| b2980 | *glcC* | GlcC transcriptional dual regulator | -7.904 | 6.69E-03 |
| b3576 | *yiaL* | conserved protein | -7.844 | 6.50E-03 |
| b4264 | *idnR* | IdnR-5-ketogluconate DNA-binding transcriptional dual regulator | -7.806 | 4.72E-05 |
| b0598 | *cstA* | peptide transporter induced by carbon starvation | -7.796 | 2.34E-06 |
| b0677 | *nagA* | N-acetylglucosamine-6-phosphate deacetylase | -7.779 | 2.91E-06 |
| b3565 | *xylA* | xylose isomerase | -7.759 | 8.45E-05 |
| b2841 | *araE* | AraE arabinose MFS transporter | -7.702 | 3.29E-05 |
| b3722 | *bglF* | β-glucoside PTS permease | -7.674 | 2.91E-03 |
| b4122 | *fumB* | fumarase B | -7.666 | 4.76E-03 |
| b3451 | *ugpE* | glycerol-3-phosphate / glycerol-2-phosphate ABC transporter - putative membrane subunit | -7.645 | 3.68E-03 |
| b0593 | *entC* | isochorismate synthase 1 | -7.627 | 1.46E-03 |
| b3117 | *tdcB* | catabolic threonine dehydratase | -7.625 | 3.49E-03 |
| b4138 | *dcuA* | DcuA dicarboxylate Dcu transporter | -7.567 | 2.22E-04 |
| b2171 | *yeiP* | predicted dehydrogenase, NAD-dependent | -7.564 | 3.16E-06 |
| b4323 | *uxuB* | D-mannonate oxidoreductase | -7.543 | 2.95E-04 |
| b3567 | *xylG* | xylose ABC transporter - ATP binding subunit | -7.532 | 1.64E-03 |
| b4118 | *melR* | MelR DNA-binding transcriptional dual regulator | -7.532 | 3.25E-04 |
| b3392 | *hofP* | protein involved in utilization of DNA as a carbon source | -7.480 | 4.16E-04 |
| b0584 | *fepA* | FepA, outer membrane receptor for ferric enterobactin (enterochelin) and colicins B and D | -7.455 | 9.11E-04 |
| b4068 | *yjcH* | conserved inner membrane protein | -7.439 | 5.70E-03 |
| b3577 | *yiaM* | predicted transporter | -7.422 | 9.06E-03 |
| b4069 | *acs* | acetyl-CoA synthetase (AMP-forming) | -7.410 | 2.41E-03 |
| b2509 | *xseA* | exonuclease VII, large subunit | -7.369 | 3.53E-04 |
| b2703 | *srlE* | glucitol/sorbitol-specific enzyme IIB component of PTS | -7.357 | 3.56E-03 |
| b3564 | *xylB* | xylulokinase | -7.355 | 5.71E-03 |
| b4265 | *idnT* | L-idonate / 5-ketogluconate / gluconate transporter | -7.343 | 2.11E-05 |
| b4030 | *psiE* | predicted phosphate starvation-inducible protein | -7.315 | 3.38E-03 |
| b0756 | *galM* | galactose-1-epimerase | -7.298 | 1.11E-03 |
| b4476 | *gntU* | GntU gluconate Gnt transporter | -7.276 | 4.15E-06 |
| b0726 | *sucA* | 2-oxoglutarate decarboxylase, thiamin-requiring | -7.275 | 5.58E-04 |
| b0431 | *cyoB* | cytochrome bo terminal oxidase subunit I | -7.263 | 5.70E-05 |
| b4239 | *treC* | trehalose-6-phosphate hydrolase | -7.247 | 3.92E-03 |
| b4004 | *zraR* | ZraR transcriptional activator | -7.224 | 5.09E-03 |
| b3418 | *malT* | MalT transcriptional activator | -7.209 | 7.40E-04 |
| b0109 | *nadC* | quinolinate phosphoribosyltransferase | -7.164 | 4.04E-07 |
| b2708 | *gutQ* | D-arabinose 5-phosphate isomerase | -7.155 | 5.02E-03 |
| b4268 | *idnK* | D-gluconate kinase, thermosensitive | -7.151 | 2.58E-04 |
| b3574 | *yiaJ* | YiaJ DNA-binding transcriptional repressor | -7.150 | 2.97E-05 |
| b3241 | *aaeA* | AaeAB Hydroxylated, Aromatic Carboxylic Acid Efflux Transport System Protein A | -7.148 | 2.86E-03 |
| b4240 | *treB* | fused trehalose(maltose)-specific PTS enzyme: IIB component/IIC component | -7.107 | 5.59E-04 |
| b4124 | *dcuR* | DcuR transcriptional activator | -7.106 | 2.41E-04 |
| b3135 | *agaA* | predicted truncated N-acetylgalactosamine-6-phosphate deacetylase | -7.102 | 3.47E-03 |
| b3749 | *rbsA* | ribose ABC transporter - putative ATP binding subunit | -7.095 | 8.54E-04 |
| b4015 | *aceA* | isocitrate lyase | -7.045 | 5.26E-03 |
| b3582 | *sgbU* | predicted L-xylulose 5-phosphate 3-epimerase | -7.021 | 2.47E-03 |
| b3395 | *hofM* | protein involved in utilization of DNA as a carbon source | -7.021 | 5.26E-05 |
| b3133 | *agaV* | PTS system, cytoplasmic, N-acetylgalactosamine-specific IIB component 2 (EIIB-AGA) | -6.991 | 2.69E-03 |
| b3093 | *exuT* | ExuT hexuronate MFS transporter | -6.960 | 8.08E-04 |
| b3092 | *uxaC* | D-glucuronate isomerase / D-galacturonate isomerase | -6.958 | 2.71E-03 |
| b4003 | *zraS* | ZraS sensory histidine kinase | -6.938 | 8.31E-03 |
| b0192 | *nlpE* | outer membrane lipoprotein NlpE, involved in surface sensing | -6.874 | 3.30E-05 |
| b4287 | *fecE* | ferric dicitrate ABC transporter - ATP binding subunit | -6.829 | 3.35E-03 |
| b3118 | *tdcA* | TdcA DNA-binding transcriptional activator | -6.825 | 1.44E-05 |
| b1205 | *ychH* | stress-induced protein | -6.742 | 5.99E-04 |
| b4119 | *melA* | α-galactosidase | -6.720 | 9.40E-03 |
| b3214 | *gltF* | periplasmic protein | -6.710 | 2.06E-03 |
| b0597 | *entH* | proofreading thioesterase in enterobactin biosynthesis | -6.658 | 2.99E-06 |
| b3404 | *envZ* | EnvZ sensory histidine kinase | -6.652 | 2.00E-03 |
| b1656 | *sodB* | superoxide dismutase (Fe) | -6.649 | 1.37E-07 |
| b3240 | *aaeB* | AaeAB Hydroxylated, Aromatic Carboxylic Acid Efflux Transport System Protein B | -6.627 | 7.86E-03 |
| b0678 | *nagB* | glucosamine-6-phosphate deaminase | -6.617 | 5.43E-04 |
| b4233 | *mpl* | UDP-N-acetylmuramate:L-alanyl-γ-D-glutamyl-meso-diaminopimelate ligase | -6.569 | 2.75E-03 |
| b3752 | *rbsK* | ribokinase | -6.545 | 8.66E-03 |
| b3437 | *gntK* | D-gluconate kinase, thermostable | -6.538 | 2.56E-03 |
| b0429 | *cyoD* | cytochrome bo terminal oxidase subunit IV | -6.488 | 1.08E-05 |
| b0118 | *acnB* | bifunctional aconitate hydratase 2 and 2-methylisocitrate dehydratase | -6.476 | 2.65E-05 |
| b3076 | *ebgA* | evolved β-D-galactosidase, α subunit | -6.442 | 9.11E-03 |
| b2943 | *galP* | GalP - galactose MFS transporter | -6.413 | 8.71E-04 |
| b3581 | *sgbH* | 3-keto-L-gulonate 6-phosphate decarboxylase | -6.413 | 2.03E-02 |
| b4014 | *aceB* | malate synthase A | -6.377 | 1.69E-03 |
| b3424 | *glpG* | intramembrane serine protease GlpG | -6.357 | 5.73E-03 |
| b3751 | *rbsB* | ribose ABC transporter - putative periplasmic binding protein | -6.311 | 3.13E-04 |
| b3413 | *gntX* | protein involved in utilization of DNA as a carbon source | -6.248 | 7.20E-03 |

^a^From the EcoCyc database (http://ecocyc.org)

^b^Fold-change in the gene expression level between A2 and the control (average of duplicate experiments). Statistics were calculated from duplicate samples.
